# Spatially resolved fluorescence of caesium lead halide perovskite supercrystals reveals quasi-atomic behavior of nanocrystals

**DOI:** 10.1038/s41467-022-28486-3

**Published:** 2022-02-16

**Authors:** Dmitry Lapkin, Christopher Kirsch, Jonas Hiller, Denis Andrienko, Dameli Assalauova, Kai Braun, Jerome Carnis, Young Yong Kim, Mukunda Mandal, Andre Maier, Alfred J. Meixner, Nastasia Mukharamova, Marcus Scheele, Frank Schreiber, Michael Sprung, Jan Wahl, Sophia Westendorf, Ivan A. Zaluzhnyy, Ivan A. Vartanyants

**Affiliations:** 1 Deutsches Elektronen-Synchrotron DESY, Notkestraße 85, 22607 Hamburg, Germany; 2 Institut für Physikalische und Theoretische Chemie, Universität Tübingen, Auf der Morgenstelle 18, 72076 Tübingen, Germany; 3 Max Planck Institute for Polymer Research, Ackermannweg 10, 55128 Mainz, Germany; 4 Center for Light-Matter Interaction, Sensors & Analytics LISA+, Universität Tübingen, Auf der Morgenstelle 15, 72076 Tübingen, Germany; 5 Institut für Angewandte Physik, Universität Tübingen, Auf der Morgenstelle 10, 72076 Tübingen, Germany; 6 National Research Nuclear University MEPhI (Moscow Engineering Physics Institute), Kashirskoe shosse 31, 115409 Moscow, Russia

**Keywords:** Electronic materials, Quantum dots

## Abstract

We correlate spatially resolved fluorescence (-lifetime) measurements with X-ray nanodiffraction to reveal surface defects in supercrystals of self-assembled cesium lead halide perovskite nanocrystals and study their effect on the fluorescence properties. Upon comparison with density functional modeling, we show that a loss in structural coherence, an increasing atomic misalignment between adjacent nanocrystals, and growing compressive strain near the surface of the supercrystal are responsible for the observed fluorescence blueshift and decreased fluorescence lifetimes. Such surface defect-related optical properties extend the frequently assumed analogy between atoms and nanocrystals as so-called quasi-atoms. Our results emphasize the importance of minimizing strain during the self-assembly of perovskite nanocrystals into supercrystals for lighting application such as superfluorescent emitters.

## Abstract (web summary)

By utilizing spatially resolved fluorescence (-lifetime) measurements and high precision X-ray nanodiffraction, the authors correlate the influence of structural misalignment and fluorescence (-lifetime) properties of all-inorganic CsPbX_3_ (X^–^ = Br^–^, Cl^–^) perovskite superlattices.

## Introduction

Advances in the self-assembly of colloidal nanocrystals (NCs) from solution into three-dimensional arrays with long-range order have enabled the design of microscopic “supercrystals” that approach the structural precision of atomic single crystals^1^. The individual NCs, which are the building blocks of a supercrystal, are often regarded as “artificial atoms”, and hence analogies between atomic crystals and such supercrystals have been made^2,3^. NC supercrystals are susceptible to doping^4^, and they can exhibit exceptional mechanical properties^5^, quasicrystal formation^2^, enhanced electronic coupling^6^, and engineered phonon modes^7^. In view of the recent progress in exploiting the massive structural coherence in NC supercrystals to generate collective optoelectronic properties^8–10^, a critical question remains whether this artificial atom analogy can be extended towards the optical properties of NC supercrystals. Due to surface dangling bonds and surface reconstruction, even the purest and most carefully prepared atomic crystals are not structurally perfect^11,12^. For atomic crystals, such surface defects strongly affect the fluorescence spectra, lifetime, and quantum yield^13–17^. For supercrystals, this is much less understood.

In this work, we show that in close analogy to atomic crystals^18,19^, CsPbBr_2_Cl and CsPbBr_3_ NC supercrystals exhibit structural distortions near their surfaces which significantly alter their fluorescence properties. This finding is of high relevance for the application of these materials as tunable, bright emitters with superfluorescent behavior^8–10^. Superfluorescence is a key property for the design of spectrally ultra-pure laser sources^20^ or highly efficient light-harvesting systems^21^. Recent quantum chemical simulations have suggested that structural disorder in CsPbBr_3_ supercrystals and its effect on the thermal decoherence plays a pivotal role in the efficiency of the superfluorescence^22^. Previous structural investigations of ensembles of CsPbBr_3_ supercrystals by grazing-incidence small angle X-ray scattering (SAXS) indicated a primitive unit cell with slight tetragonal distortion^23^, and wide-angle X-ray scattering (WAXS) showed a high degree of structural coherence^24^. Electron microscopy of individual supercrystals revealed a frequent occurrence of local defects in the supercrystals, such as isolated NC vacancies^25^. Confocal fluorescence microscopy of individual CsPbBr_3_ supercrystals displayed spatial variations in the fluorescence peak wavelength and intensity, indicating that local structural inhomogeneities may substantially affect the fluorescence properties of the entire supercrystal^26^. Our approach is based on simultaneous WAXS and SAXS measurements with a nano-focused beam to probe the structural defects and crystallographic orientation of the supercrystal and the constituting NCs on a local level with dimensions of ~3 µm and 7–9 nm, respectively^27–29^. By correlation with diffraction-limited confocal fluorescence microscopy and modeling with density functional theory (DFT) we present proof that compressive strain, a loss of structural coherence and an increasing atomic misalignment between adjacent nanocrystals at the edges of CsPbBr_2_Cl NC supercrystals are responsible for a blueshifted emission and decrease of the fluorescence lifetimes.

## Results

**Fig. 1:**
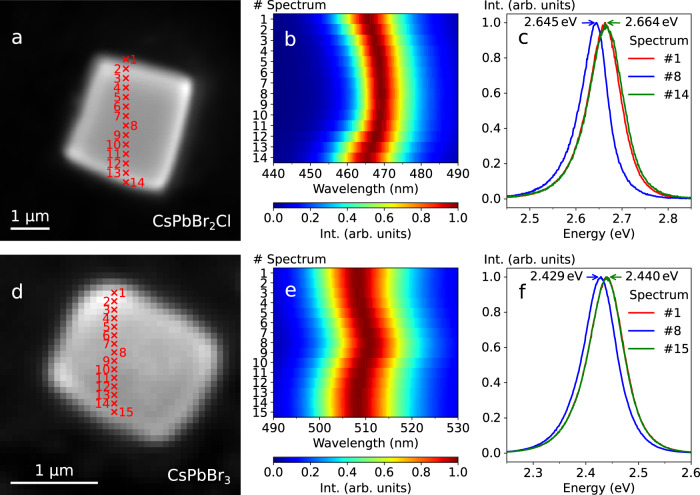
Spatially resolved fluorescence. **a** Optical micrograph of a CsPbBr_2_Cl NC supercrystal. Positions of the measured photoluminescence spectra are indicated. **b** The corresponding normalized spectra. **c** Selected normalized spectra, acquired at the edges and the center of the supercrystal. **d**–**f** Corresponding data for a CsPbBr_3_ supercrystal.

We study self-assembled CsPbBr_2_Cl and CsPbBr_3_ NC supercrystals on glass substrates (see “Methods” for details on synthesis and self-assembly of NCs). Spatially resolved photoluminescence spectra of the NC supercrystals under 405 nm excitation in a confocal laser scanning microscope with a step size of 250 nm and 100 nm, respectively, are shown in Fig. 1. When approaching an edge of the supercrystal, we find a continuous blueshift of the emission peak wavelength. This blueshift is strongest for relatively small (few µm edge length) and highly faceted supercrystals, where it reaches up to 20 meV for CsPbBr_2_Cl. We observe the same blueshifting behavior for supercrystals composed of CsPbBr_3_ NCs, although to a lesser extent (up to 12 meV).

**Fig. 2:**
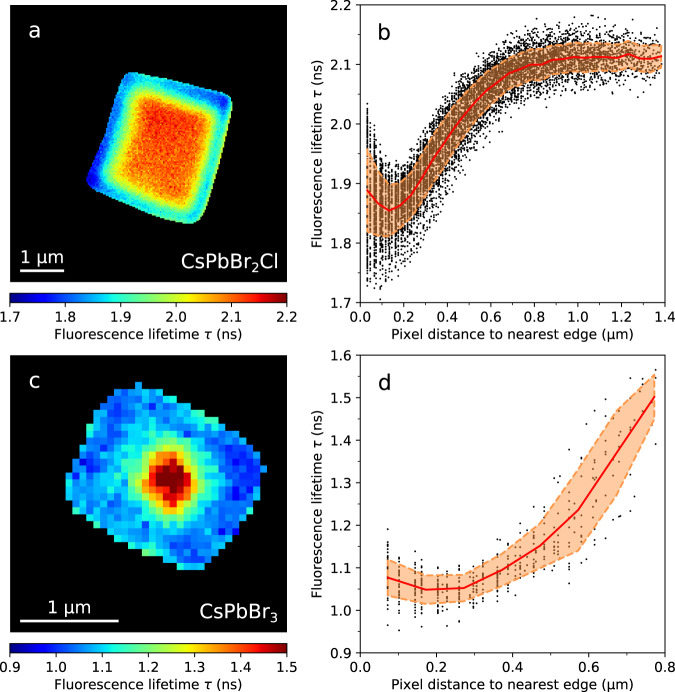
Spatially resolved fluorescence lifetime imaging. **a** Fluorescence lifetime τ image of a CsPbBr_2_Cl NC supercrystal obtained by fitting the experimental time-resolved fluorescence with a monoexponential decay function. **b** Fluorescence lifetime values τ obtained at each pixel inside the supercrystal as a function of the distance to the nearest edge, where the red line shows the mean value, and the dashed lines indicate the confidence interval of ±*σ*. **c**, **d** Analogous results for a CsPbBr_3_ NC supercrystal.

In Fig. 2, we display fluorescence lifetime images of self-assembled CsPbBr_2_Cl and CsPbBr_3_ supercrystals measured on glass substrates with a lateral resolution of 200 nm under 405 nm excitation. For both supercrystal compositions, we obtain good fits of the experimental time-resolved fluorescence by pixel-by-pixel monoexponential reconvolution using an instrument response function acquired on a clean glass coverslip (Supplementary Figs. 2, 3). In the case of supercrystals composed of CsPbBr_2_Cl NCs, we measure typical fluorescence lifetimes (τ) around 2.1 ns in the center which decrease by approximately 20% when scanning from the center of a supercrystal towards its edges. Supercrystals composed of CsPbBr_3_ NCs exhibit typical lifetime values around 1.5 ns in the center, which shorten by approximately 30% when approaching the edges. We note that this holds true only for freshly prepared NC supercrystals. After several days of exposure to air, the trend in the spatially resolved τ-values is reversed in that such aged supercrystals exhibit longer lifetimes at the edges. However, the overall blueshift of the fluorescence peak wavelength towards the edges is preserved.

**Fig. 3:**
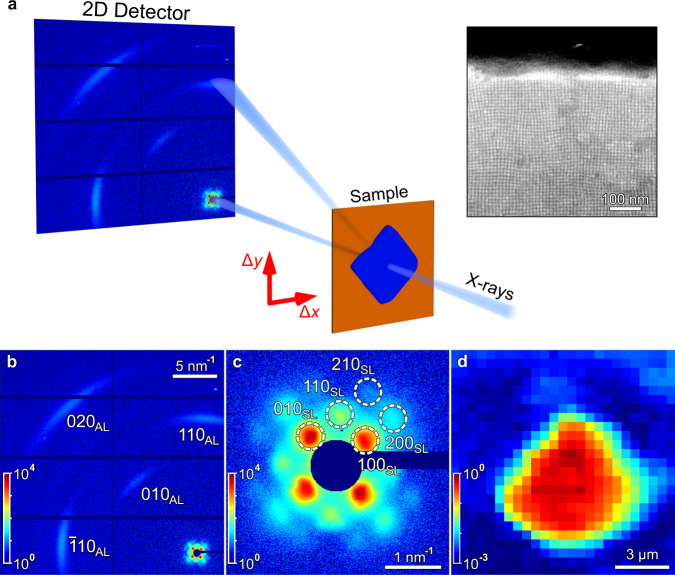
Spatially resolved X-ray nanodiffraction experiment and average diffraction patterns. **a** Scheme of the X-ray experiment. EIGER X 4 M 2D detector is positioned downstream from the sample. The arrows show the directions Δ*x* and Δ*y* of spatial scanning. Inset (top right): a SEM micrograph of the CsPbBr_2_Cl NC supercrystal. **b** Average diffraction pattern for a supercrystal. Several orders of WAXS and SAXS Bragg peaks from the atomic and supercrystal structure, respectively, are well visible. The WAXS Bragg peaks are indexed using pseudocubic notation. **c** Enlarged SAXS region of the averaged diffraction pattern. The Bragg peaks are indexed according to a simple cubic structure. **d** Diffraction map for a scan based on the integrated intensity of the SAXS diffraction patterns at *q* < 2 nm^−1^. The pixel size (the step size) is 500 nm.

To correlate the fluorescence data with the structure of the supercrystals, we carry out X-ray synchrotron measurements by SAXS and WAXS at PETRA III facility (Hamburg, Germany) (see Fig. 3a and “Methods” for details). Using a 400 × 400 nm^2^ X-ray beam, we perform a spatially resolved scan of a typical CsPbBr_2_Cl NC supercrystal on a Kapton substrate. While the results presented here are for one typical supercrystal, examples of more supercrystals are provided in the Supplementary information (Supplementary Note 7). First, all individual patterns are integrated to obtain the average structure. The averaged background-corrected WAXS and SAXS diffraction patterns are shown in Fig. 3b, c, correspondingly. The signal in the WAXS region contains three orders of Bragg peaks from the atomic lattice (Fig. 3b), and the SAXS region (shown enlarged in Fig. 3c) displays several orders of Bragg peak from the supercrystal. A real-space map of the scan based on the integrated SAXS intensity at *q* < 2 nm^−1^ is shown in Fig. 3d. The map represents a square area of high intensity corresponding to a single supercrystal. For comparison, we display a scanning electron micrograph of a similar supercrystal (see the inset in Fig. 3a and Supplementary Fig. 8) from which we determine an average NC diameter of 7.3 ± 0.4 nm and an interparticle distance of 2.5 ± 0.5 nm. For strongly faceted supercrystals, the NC diameter is rather uniform over the whole crystal. For less faceted supercrystals, occasional ensembles of smaller NCs are found in the vicinity of the edges. However, the spatial extent of such smaller NC populations is always limited to ~200 nm (see Supplementary Note 3).

The average diffraction pattern in the WAXS region (see Fig. 3b) contains four prominent Bragg peaks, originating from the atomic lattice (AL) of the NCs. Their radial positions at *q* = 10.93 nm^−1^, 15.44 nm^−1^, and 21.90 nm^−1^ (see Supplementary Fig. 10) can be attributed to a cubic AL. We note that although a cubic phase for CsPbBr_2_Cl has been reported^30,31^, the most stable phase at room temperature is expected to be orthorhombic. Due to the small NC size and the resulting broadening of the Bragg peaks, it is impossible to distinguish between these two very similar structures. Thus, we use a pseudocubic notation to index the WAXS peaks: 110 and 002 orthorhombic peaks correspond to 100_AL_ pseudocubic peak, 112 and 200 – to 110_AL_, and 220, 004 – to 200_AL_ peaks. The present peaks and their azimuthal positions indicate a primary orientation of the NCs along the [001]_AL_ axis with respect to the incident beam. We find the unit cell parameter to be *a*_AL_ = 0.575 ± 0.003 nm, which is in good agreement with previously reported values for CsPbBr_2_Cl^32^. From the peak broadening, we extract the NC size (*d*) and lattice distortion (*g*) using the Williamson–Hall method with *d* = 6.8 ± 0.1 nm and *g* = 2.3 ± 0.1% (see Supplementary Note 4). The obtained NC size is in good agreement with the scanning electron microscopy (SEM) results.

The SAXS pattern in Fig. 3c represents the typical 4-fold pattern of a simple cubic lattice oriented along the [001]_SC_ axis with four visible orders of Bragg peaks that can be attributed to 100_SC_, 110_SC_, 200_SC_, and 210_SC_ reflections of the supercrystal of NCs. We determine an average unit cell parameter of *a*_SC_ = 9.9 ± 0.4 nm. Considering the NC size obtained by SEM, we obtain an interparticle distance of 2.6 ± 0.4 nm, which is in good agreement with the SEM result (2.5 ± 0.5 nm). All crystallographic axes of the NCs are aligned with the corresponding axes of the supercrystal (e.g. [100]_AL_||[100]_SC_ and [010]_AL_||[010]_SC_), which is consistent with ref. ^25^.

**Fig. 4:**
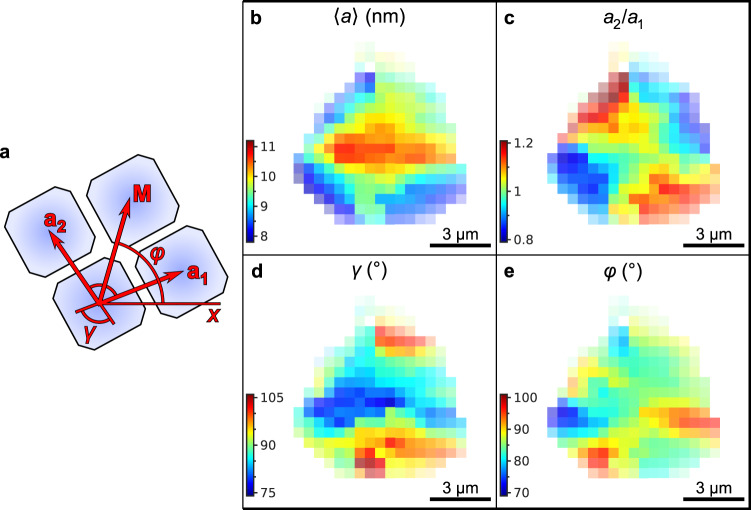
Spatially resolved SAXS. **a** Definition of the geometrical parameters of a superlattice unit cell: the basis vectors **a**_**1**_ and **a**_**2**_ with the angle *γ* between them, and the mean line **M** between the basis vectors at the angle *φ*. **b** Average unit cell parameter 〈*a*〉 = (*a*_1_+*a*_2_)/2. **c** Ratio *a*_2_/*a*_1_ of the unit cell parameters along the basis vectors **a**_**2**_ and **a**_**1**_. **d** Angle *γ* between the basis vectors **a**_**1**_ and **a**_**2**_. **e** Azimuthal Position *φ* of the mean line **M** between the basis vectors **a**_**1**_ and **a**_**2**_. The pixel size in (**b**–**e**) is 500 nm.

Analyzing individual SAXS patterns from different locations on the supercrystal, we find substantial local deviations from the average structure (see Supplementary Fig. 12 for examples of single diffraction patterns). To illustrate this, from the Bragg peak positions, we extract the basis vectors *a*_*1*_ and *a*_*2*_, the angle *γ* between them, and the average azimuthal position *φ*, which are defined in Fig. 4a (see the “Methods” section for details). As depicted in Fig. 4b, the mean unit cell parameter is largest in the center of the supercrystal with 10.7 nm and smallest at the edges with 7.8 nm. Although both unit cell parameters *a*_*1*_ and *a*_*2*_ decrease at the edges (see Supplementary Fig. 16, for separate maps of *a*_*1*_ and *a*_*2*_ values), we observe that this lattice contraction is anisotropic. The ratio of the in-plane unit cell parameters *a*_*2*_/*a*_*1*_ differs from unity by ±20% in such a way that the NC spacing in the directions along the nearest supercrystal boundary is smaller than normal to it, as shown in Fig. 4c. We note that the mean value 〈*a*〉 = 9.4 ± 0.7 nm is slightly smaller than the unit cell parameters extracted from the average diffraction pattern. We attribute this to the low intensity of scattering from the supercrystal edges, which reduces their contribution to the average pattern. We do not observe a clear trend in the size of the SAXS Bragg peaks (see Supplementary Fig. 15, for the maps). The instrumental peak broadening, determined by the incident X-ray beam size is about 0.015 nm^−1^ (full width at half maximum, FWHM). The observed peak sizes are much larger and vary in the range from 0.05 nm^−1^ to 0.2 nm^−1^ and, as such, they depend mainly on the superlattice distortion. The characteristic length scale on which this distortion evolves is, most probably, smaller than the incident beam. Thus, the areas with different lattice parameters simultaneously illuminated by the incident beam lead to the peak broadening.

The angle *γ* between the [100]_SC_ and [010]_SC_ axes differs from its average value of 〈*γ*〉 = 90 ± 6° in a range of 76° to 105° over the whole supercrystal as shown in Fig. 4d. Specifically, we find γ > 90° close to the top and bottom corners of the supercrystal and γ < 90° close to the left and right corners. Thus, the angle pointing towards the corner of the supercrystal is always obtuse. We further calculate the azimuthal position *φ* of the mean line **M** between the [100]_SC_ and [010]_SC_ axes. This angle can be interpreted as the azimuthal orientation of the unit cell of the supercrystal. The orientation changes inhomogeneously throughout the superlattice in the range from 72° to 97° as shown in Fig. 4e. There is no obvious correlation between the lattice orientation and the spatial position within the sample. Overall, these results suggest that the supercrystal is simple cubic on average, but it exhibits substantial local monoclinic distortions.

**Fig. 5:**
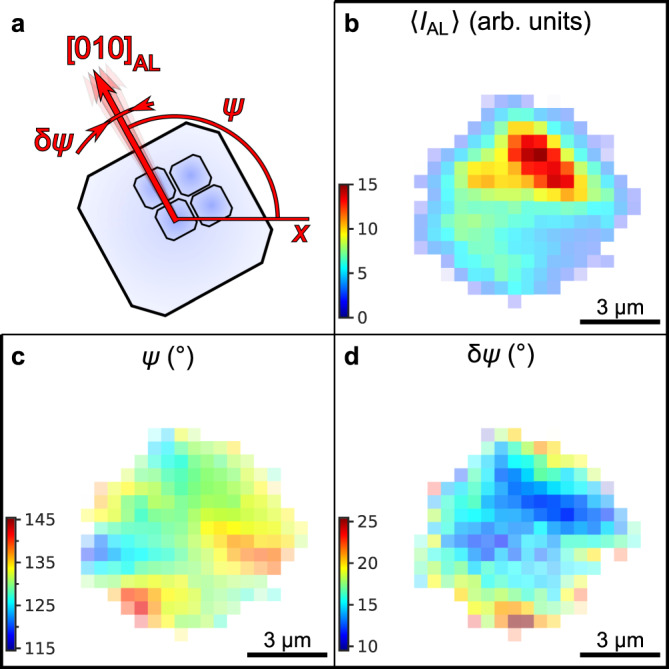
Spatially resolved WAXS. **a** Definition of the geometrical parameters of the atomic lattice extracted by fitting of the Bragg peaks. **b** Mean intensity of the WAXS Bragg peaks 〈*I*_AL_〉. **c** Azimuthal position *ψ* of the 100_AL_ crystallographic axis of the NCs. **d** FWHM *δψ* of the angular disorder of the NCs around the mean azimuthal position *ψ* extracted from the azimuthal FWHMs of the Bragg peaks by the Williamson–Hall method. The pixel size in (**b**–**d**) is 500 nm.

We analyze the Bragg peaks in the WAXS region of individual diffraction patterns at different locations to study the angular orientation of the NCs inside the superlattice. From the WAXS Bragg peak analysis, we extract the average WAXS intensity 〈*I*_AL_〉 and the azimuthal position *ψ* of the [010]_AL_ axis defined in Fig. 5a (see “Methods” section and Supplementary Note 6). In contrast to the intensity of the SAXS Bragg peaks, the WAXS intensity 〈*I*_AL_〉 decreases towards the edges, as shown in Fig. 5b, indicating an out-of-plane rotation of the NCs that shifts the Bragg peaks slightly out of the Ewald sphere^28^. We find that *ψ* changes in a wide range from 120° to 142° as shown in Fig. 5c. The map of *ψ* resembles that of the azimuthal orientation *φ* of the mean line **M**, shown in Fig. 4e. The 45° offset between the [010]_AL_ axis and the mean line **M** indicates the alignment of the [110]_AL_ axis with the mean line **M** between the [100]_SC_ and [010]_SC_ axes (see Supplementary Fig. 23).

From the azimuthal FWHMs of the WAXS Bragg peaks, we extract the angular disorder *δψ* of the individual nanocrystals at each spatial point by the Williamson–Hall method as shown in Fig. 5d (see “Methods” for details). The disorder is smallest in the center of a supercrystal (9.9°) and increases to a maximum of 24.0° at the edges. The mean value of the angular disorder is 〈*δψ*〉 = 16.1 ± 2.8°, which is consistent with previously observed values for similar superstructures^25,27–29^.

**Fig. 6:**
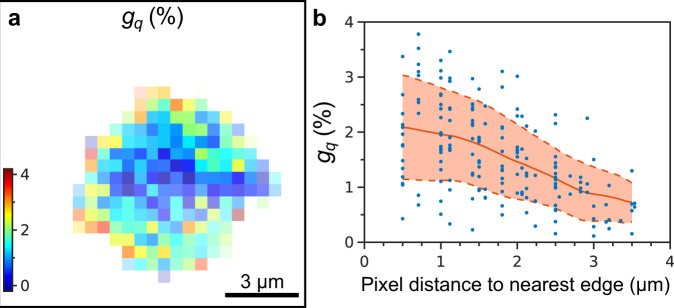
Spatially resolved atomic lattice distortion. **a** Atomic lattice distortion *g*_*q*_ extracted from the radial FWHMs of the WAXS Bragg peaks by the Williamson–Hall method. The pixel size is 500 nm. **b** The same value *g*_*q*_ for each pixel plotted against the distance from this pixel to the nearest edge of the supercrystal. The red line shows the mean value, the dashed lines indicate the confidence interval of ±*σ*.

Despite the fact that the atomic lattice parameter *a*_AL_ is constant within the error bars throughout the whole supercrystal (see Supplementary Fig. 20, for the map of *a*_AL_), we find a difference in the radial width of the Bragg peaks at different locations. By the Williamson–Hall method, we extract the lattice distortion *g*_*q*_ (the ratio *δa*_AL_/*a*_AL_, where *δa*_AL_ is the FWHM of the unit cell parameter distribution around the mean value *a*_AL_) at each spatial point (see “Methods” section for details). We find a clear trend of increasing atomic lattice distortion towards the edges of the supercrystal with a maximum of 2% at the edge, while it is about 1% at a distance 3 µm into the center, as shown in Fig. 6. The trend is even more evident for another supercrystal with particularly good signal-to-noise ratio of the WAXS intensity (see Supplementary Fig. 29).

**Fig. 7:**
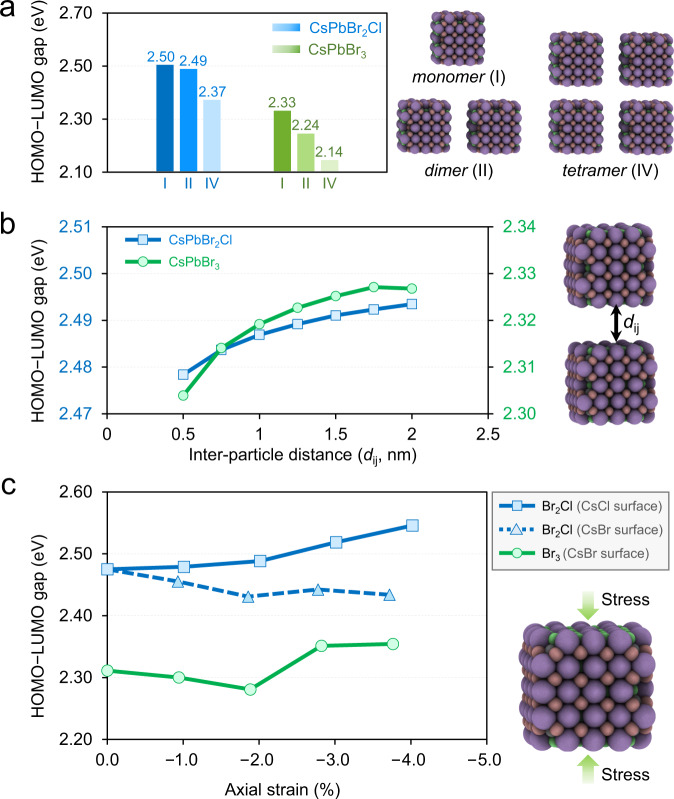
Density Functional Modeling. Computed HOMO–LUMO gaps as a function of (**a**) number of neighboring particles considered (dimers and tetramers are 0.5 nm apart), (**b**) distance *d*_*ij*_ between two adjacent particles, and (**c**) applied axial strain, for both CsPbBr_2_Cl and CsPbBr_3_ particles. All energies are in eV computed at PBE/DZVP level of theory.

To rationalize the experimental trend of increased fluorescence energies at the edges of the supercrystal as compared to its center, we carry out density functional modeling of the system. We consider three individual contributions in this regard. First, we recognize that the number of nearest neighbors at the surface of the supercrystal is lower than that in the center, leading to stronger exciton confinement and hence increased fluorescence energies at the edges. Indeed, our DFT calculations confirm this trend in Fig. 7a, which is consistent with the blueshift of the fluorescence spectra observed experimentally for the NCs at the edges. “Nearest neighbors” refers here to adjacent NCs with near-perfect orientational order, that is, a low value of *δψ* (Fig. 5d). A large orientational misalignment (*δψ*) is likely to have a similar effect on nearest-neighbor coupling as a reduced number of nearest neighbors. Second, we anticipate that the shorter interparticle spacing (Fig. 4b) should facilitate better electronic coupling between the nanocrystals at the edges and, therefore, a decrease in the optical gap at the edges is anticipated. While this expectation is confirmed computationally in Fig. 7b, we note that it is exactly opposite to what is observed experimentally in Fig. 1b, e (see Discussion section for details). Third, the supercrystal is compressed at the edges, as evident from Fig. 4b. While it is reasonable to assume that the compressive strain will mostly manifest in a denser packing of the soft oleylamine/oleic acid ligand sphere of the NCs, we also consider a partial compression of the hard-inorganic lattice-core. In Fig. 7c we calculate the effect of such compression on the HOMO–LUMO gap (*E*_gap_) of the NC. While axial stress applied to the CsCl-terminated surface of the CsPbBr_2_Cl particle results in a steady increase of the optical gap consistent with the experiment, similar stress on the CsBr-terminated surfaces of both particles are found to both increase or decrease *E*_gap_, depending on the magnitude of the applied stress.

Overall, our computational modeling suggests that the spectral blueshift of the fluorescence from the edges of the supercrystal can be caused mainly due to a reduced NC coordination number at the edges as well as the compressive atomic lattice strain in some cases, knowing that the third factor—the shorter interparticle distance—works in the opposite direction, facilitating electronic coupling between adjacent nanocrystals and decreasing the optical gap. However, since the experimentally measured spectral shift is seemingly a combination of all three effects discussed above, a fully quantitative prediction would require more detailed knowledge on their relative contributions as well as the relative orientation and positions of individual nanocrystals, which are currently not available.

## Discussion

When NCs are self-assembled into supercrystals from colloidal solution via slow drying, the increasing curvature and surface tension of the evaporating solvent invokes compressive strain on the supercrystal^33,34^. We hold such a strain responsible for the observed compression of the unit cell parameter by over 20% of the CsPbBr_2_Cl NC supercrystals in Fig. 4b. This compression is possible due to the softness of the oleylamine/oleic acid ligand shell of the NCs, enabling a large decrease of the interparticle distance by growing interdigitation of adjacent ligand spheres. We note that the compression occurs gradually over a length scale of many lattice planes (>1 µm), meaning that it is not a localized surface reconstruction as commonly observed in atomic crystals^12^. The accompanying loss in the angular correlation of the constituting NCs with the superlattice fits a scenario where strain in the supercrystal is partially relieved by forming local structural defects. The comparison of the average (Fig. 3c) *vs*. the local (Fig. 4) structure of the supercrystal shows that such distortions are indeed frequently present. We note that recent work on CsPbBr_3_ NC supercrystals reported perfect structural coherence exclusively in the out-of-plane direction^24^. Since our experiment is only sensitive to in-plane structural features, the findings here are not contradictive to that report.

Our results in Fig. 4c support the view of Kapuscinsky et al. that strain during the self-assembly is initially isotropic but later becomes increasingly anisotropic^33^. In a simple cubic supercrystal, the preferred direction for anisotropic structural changes to manifest is the 〈111〉_SC_, which will result in a shear deformation of the ligand spheres^35^. The expected structure of the supercrystal after this shear deformation is reasonably resembled by the local structure depicted in Fig. 4.

The compression in the supercrystals is not exclusively limited to the soft ligand sphere. With an interparticle distance of <1 nm close to the edges of a supercrystal, the space for the two ligand spheres of adjacent NCs is so constrained, that the inorganic cores of the NCs become compressed as well (Fig. 6). Strain in lead halide perovskite thin films plays an important role for their optoelectronic properties and application in photovoltaic devices^36^. Our fluorescence and fluorescence lifetime data in Figs. 1, 2 suggest that this is also the case for lead halide NC supercrystals. A comparison of Fig. 1 with Fig. 4b reveals a strong correlation between the gradual blueshift of the fluorescence peak wavelength and the progressive compression of the supercrystal. We suggest that the shift by up to 20 meV is the result of three, partially competing phenomena: (1) a loss in structural coherence as well as isoorientation of NCs (Fig. 5c, d), (2) a decrease of the interparticle distance (Fig. 4b), and (3) the distortion of the atomic lattices of the NCs (Fig. 6). Our DFT calculations in Fig. 7a suggest that the first effect should be associated with a significant blueshift of the fluorescence due to reduced coupling, consistent with a previous report about the importance of structural coherence for electric transport in supercrystals^6^. While the second effect can only lead to a redshift (Fig. 7b), the third effect is also shown to invoke a blueshift for specific facets or magnitudes of strain (Fig. 7c).

With reference to several studies on CsPbBr_3_ NCs which reported a redshifted fluorescence after assembly into supercrystals, we note that the resultant peak wavelength may further be affected by the concomitant changes in the dielectric environment, aging, miniband formation as well as cooperative emission^8,9,37–39^. However, most of these observations were made under markedly different conditions, such as low temperature, prolonged exposure to air, or self-assembly at the liquid/gas interface, which may be the reason that they are not a dominating factor in our study.

We note a previous report on the spatially resolved fluorescence of CsPb(I_0.28_Br_0.72_)_3_ NC supercrystals with a similar fluorescence blueshift between the center and the edge^26^. As a main conclusion, gradual release of I_2_ gas under intense laser illumination led to the blueshift since lead bromide perovskites exhibit a larger bandgap than the corresponding lead iodide perovskites. The authors argued that the I_2_ loss commenced from the edges towards the center, which would explain the spatial fluorescence variations. In CsPbBr_2_Cl however, this mechanism is not easily applicable since the reduction potential of Br^–^ is much lower than that of I^–^. In line with this, CsPbBr_3_ NC supercrystals without a halide mixture show a similar blueshift, indicating that a change in the mixed halide composition is not required to observe the effects reported here.

The decrease of the fluorescence lifetime in Fig. 2 is also strongly correlated with the gradual compression of the supercrystal towards the edges. Moreover, many supercrystals exhibit particularly decreased lifetime values at the corners, which bears similarities with the anisotropic changes in the lattice spacings in Fig. 4c, highlighting again the correlation between structural and optical properties. We speculate that the increased atomic lattice distortion and loss of structural coherence near the edges of the supercrystals result in a reduced stability of the excited state of the emitting NCs. This view is supported by the decreased radiative lifetime values from these locations as well as previous reports on the fluorescence lifetime at grain boundaries of large organic-inorganic pervoskites^40,41^. In view of the currently pursued application of lead halide NC supercrystals as superfluorescent emitters^8,10^, this would imply that bright and coherent emission originates from the center of the supercrystals as long as they are freshly prepared. Conversely, for aged CsPbBr_2_Cl NC supercrystals, the lifetimes are longest at the edges, which points to an increased stability of the excited state, potentially due to the formation of a protective oxide shell^42^.

As an alternative explanation for the spatial differences in the fluorescence (-lifetime) in the supercrystals, we also consider a photon propagation effect, that is, multiple emission and (re-)absorption events, which become more likely with increasing thickness of the emitter material^37^. Therefore, reabsorption should occur predominantly in the center of the supercrystals but not at the edges. This effect results in an overall redshift of the fluorescence and an increase of the fluorescence lifetime, which would be in line with the observations in this work^43,44^. Moreover, we would expect the absolute fluorescence intensity per emitter to be lower for an area with frequent reabsorption events and the time-resolved fluorescence decay to be increasingly multiexponential due to the non-radiative losses and multi-step nature of the photon propagation effect. However, we find the fluorescence decay to be monoexponential (Supplementary Figs. 2c, 3c) and the fluorescence intensity to be highest in the center, from where it gradually decreases toward the edges (Supplementary Figs. 6b, 7b). This decrease extends over a much larger distance than the flattening of the edges which we occasionally observe on less facetted supercrystals (Supplementary Fig. 9c), such that the high fluorescence intensity in the center cannot be a mere thickness effect. We note that we would expect a negative correlation between the fluorescence intensity with its corresponding lifetime if reabsorption was dominant in the supercrystals, but we do not find such a correlation in our data (Supplementary Figs. 6f, 7f). In summary, given the relatively high fluorescence quantum yield of lead halide perovskite NCs, reabsorption is likely to partially contribute to the spatially varying optical properties of supercrystals thereof^45^, but our data is inconsistent with it as the dominant cause.

In conclusion, supercrystals of lead halide perovskite NCs self-assembled from solution exhibit a loss in structural coherence, an increasing atomic misalignment between adjacent NCs, and compressive strain near their surfaces. These structural distortions are strongly correlated with a blueshifted fluorescence and decreased radiative lifetimes. We note that structural distortion and surface defects have been shown to strongly affect the fluorescence properties in atomic crystals, such as transition metal dichalcogenides^13–17^. The structure-fluorescence correlations in supercrystals revealed here are thus another example for the analogy between atoms and NCs as so-called quasi-atoms.

## Methods

### Chemicals

1-Octadecene (ODE), technical grade, 90%, Sigma Aldrich; Oleic acid (OA), 97%, Acros Organics; Oleylamine (OAm), 80–90%, Acros Organics; Cesium carbonate (Cs_2_CO_3_), 99.99% (trace metal basis), Acros Organics; Lead(II)chloride (PbCl_2_), 99.999% (trace metal basis), Sigma Aldrich; Lead(II)bromide (PbBr_2_), ≥98%, Sigma Aldrich; Toluene, 99.8%, extra dry, AcroSeal, Acros; Tetrachloroethylene (TCE), ≥99%, Acros Organics; Kapton® polyimide membranes (125 μm thickness) were purchased from DuPont; Si/SiOx wafers (200 nm SiO_*x*_ thickness) were purchased from Siegert Wafer GmbH. All chemicals were used as purchased.

### Preparation of Cs-oleate

203.5 mg Cs_2_CO_3_ (0.625 mmol) was loaded into a 25 mL three-neck flask along with 10 mL 1-octadecene and 0.625 mL oleic acid, dried for 1 h at 120 °C and then heated to 150 °C under nitrogen atmosphere until all Cs_2_CO_3_ reacted with oleic acid. The mixture was kept in a glovebox and heated to 110 °C before injection.

### Synthesis of CsPbX_3_ nanocrystals

CsPbX_3_ NCs were made by a hot-Injection synthesis using a modified literature method^46^. To synthesize 9 nm CsPbBr_3_ or 7 nm CsPbBr_2_Cl NCs, 138 mg (0.38 mmol) PbBr_2_ or 92 mg (0.25 mmol) PbBr_2_ and 35 mg (0.125 mmol) PbCl_2_ were degassed in 10 mL ODE in a 25 mL three-neck flask under reduced pressure at 120 °C for 2 h. Then, 1 mL of dried oleylamine (OAm) and 0.5 mL of dried oleic acid (OA) were injected at 120 °C under nitrogen atmosphere with continuous stirring and the reaction mixture was heated to 160 °C. After the solubilization was completed, 0.8 mL of a previously prepared solution of Cs-oleate in ODE (0.125 M) was swiftly injected, and the reaction mixture was cooled to room temperature using an ice-bath.

### Isolation and purification of CsPbX_3_ nanocrystals

CsPbX_3_ NCs were collected by centrifuging the suspension (4650 *g*, 10 min), decanting the supernatant, and collecting the precipitate. The precipitate was centrifuged again without addition of a solvent (4650 g, 5 min), and the resulting supernatant was removed with a syringe, to separate the traces of residual supernatant. The precipitate was dissolved in 2 mL hexane and centrifuged again (590 *g*, 5 min) to remove aggregates and larger particles. The resulting supernatant was filtered through a 0.2 µm PTFE syringe filter and stored as stock solution inside of a glovebox with a typically concentration of 16 mM following Maes et al.^47^.

### Self-assembly of NC superlattices

For the growth of supercrystals, different substrates (Si wafer, Kapton, glass) were used, depending on the desired experiment. The self-assembly experiment was set up in a glass Petri dish (with a 60 mm diameter), for this purpose three substrates each were placed in such a Petri dish together with a PTFE-lid filled with 1 mL tetrachloroethylene. To each of these substrates, 40 µL of a 1–3 mM solution of the perovskites in TCE was added. The lid of the Petri dish was closed, covered with aluminum foil, and allowed to stand for 24 h. After that, the lid was opened and left for another 5 h to dry completely. All self-assembly preparations were performed under inert atmosphere. The more monodisperse the size distribution of the perovskites, the better the resulting superlattices

### Spatially resolved optical measurements

All spatially resolved optical measurements were performed using a home-built inverted confocal laser scanning microscope. The measurements were performed on glass substrates utilizing a high numerical aperture oil immersion objective (NA = 1.4) and a 405 nm pulsed diode laser (Picoquant LDH P-C-405) with variable repetition rates (Picoquant PDL 800-D laser driver) as the excitation source. Under these conditions the lateral resolution of the instrument is approximately 200 nm. A single photon avalanche diode (MPD PDM Series) was used in conjunction with the Picoquant HydraHarp 400 as a time-correlated single photon counting system to detect time-resolved fluorescence. Time-resolved data acquisition and analysis was performed using Picoquants SymPhoTime 64 software package. The spectral data was recorded using an Acton Spectra Pro 2300i spectrometer with a 300 grooves/mm grating. The detector temperature (Princeton PIXIS CCD) was kept steady at −45 °C.

### X-ray diffraction experiment

The nanodiffraction experiment was performed at the Coherence Applications beamline P10 of the PETRA III synchrotron source at DESY. An X-ray beam with the wavelength *λ* = 0.0898 nm (*E* = 13.8 keV) was focused down to a spot size of approximately 400 × 400 nm^2^ (FWHM) with a focal depth of about 0.5 mm at the GINIX nanodiffraction endstation^48^. The two-dimensional detector EIGER X 4 M (Dectris) with 2070 × 2167 pixels and a pixel size of 75 × 75 μm^2^ was positioned 412 mm downstream from the sample. The detector was aligned ~6 cm off-center in both directions normal to the incident beam to allow simultaneous detection of SAXS and WAXS. We performed a spatially resolved scan of the sample on a Kapton substrate by 25 × 25 spatial points with 500 nm step size and collected 625 diffraction patterns in transmission geometry. The exposure time was set to 0.5 s to prevent radiation damage of the sample. The background scattering pattern from a pure Kapton film was subtracted from every collected pattern.

### Bragg peak analysis

Each diffraction pattern was interpolated onto a polar coordinate grid with the origin at the direct beam position. The radial profiles were obtained by averaging along the azimuthal coordinate. To extract parameters of the WAXS and SAXS Bragg peaks separately, we fitted each of them by the 2D Gaussian function

$$I\left(q,\varphi \right)=\frac{{I}_{0}}{2\pi {\sigma }_{q}{\sigma }_{\varphi }}{{\exp }}\left[-\frac{{\left(q-{q}_{0}\right)}^{2}}{2{\sigma }_{q}^{2}}-\frac{{\left(\varphi -{\varphi }_{0}\right)}^{2}}{2{\sigma }_{\varphi }^{2}}\right],$$

where *I*_*0*_ is the integrated intensity, *q*_*0*_ and *φ*_*0*_ are the radial and azimuthal central positions, and *σ*_*q*_ and *σ*_*φ*_ are the corresponding root mean square (rms) values. The FWHMs of the Bragg peaks were evaluated according to relations: ${w}_{q}=2\sqrt{2{{{{{\rm{ln}}}}}}2}{\sigma }_{q}$ and ${w}_{\varphi }=2\sqrt{2{{{{{\rm{ln}}}}}}2}{\sigma }_{\varphi }$. The fitting was done in the appropriate region of the polar coordinates with a single isolated Bragg peak.

For the SAXS peaks, the parameters were pairwise averaged for the corresponding Friedel pairs of the Bragg peaks to improve statistics. The resulting momentum transfer values and angles were used to calculate the real-space parameters of the unit cell: the length of the basis vectors *a*_*1*_ and *a*_*2*_, the angle *γ* between them and the average azimuthal position *φ* counted counterclockwise from an arbitrary horizontal axis (see Supplementary Materials for details).

For the WAXS peaks, we calculated an average Bragg peak intensity *I*_*AL*_ and the azimuthal position *ψ* of the [010]_AL_ axis. To obtain the average azimuthal position *ψ*, we averaged all four azimuthal positions for 010_AL_, 020_AL_, 110_AL_, and $\bar{1}00$_AL_ Bragg peaks, but corrected the last two values by +45° and −45°, respectively. We used the Williamson–Hall method^49^ to analyze the size of the WAXS Bragg peaks at each spatial point of the supercrystal. The FWHM of the Bragg peak is determined by the NC size and the lattice distortion as follows:

1 $${w}_{q,\varphi }^{2}\left(q\right)={\left(\frac{2\pi K}{L}\right)}^{2}+{\left({g}_{q,\varphi }q\right)}^{2},$$

where *w*_*q,φ*_ is the FWHM of the Bragg peak at *q* in radial or azimuthal direction, respectively, *L* is the NC size, *g*_*q,φ*_ is the radial or angular lattice distortion of the atomic lattice, respectively, *K* is a dimensionless shape parameter that is about 0.86 for cubic NCs^50^. The radial lattice distortion *g*_*q*_ calculated from the radial FWHM *w*_*q*_ is equal to the ratio *δa*_AL_/*a*_AL_, where *δa*_AL_ is the FWHM of the unit cell parameter distribution around the mean value *a*_AL_. The angular lattice distortion *g*_*φ*_ calculated from the azimuthal FWHM *w*_*φ*_ is equal to the FWHM *δψ* of the angular distribution of the NCs around their average azimuthal position *ψ*. For the spatially resolved analysis of the FWHMs, the NC size *L* was fixed at the value, obtained from the average radial profiles. For details of the analysis, see Supplementary materials.

### Scanning electron and atomic force microscopy

SEM imaging of supercrystals on Si/SiO_x_ devices was performed with a HITACHI model SU8030 at 30 kV. To estimate the thickness of micro-crystals, samples were titled by 45° with respect to the incoming electron beam. AFM investigations were conducted with a Bruker MultiMode 8 HR in contact mode.

### Density functional theory calculations

All computations are performed using the CP2K 5.1 program suite using the Quickstep module^51^. The PBE exchange correlation functional^52^, a dual basis of localized Gaussians and plane waves (GPW)^53^ with a 350 Ry plane-wave cutoff, double-ζ basis-set augmented with polarization functions (MOLOPT variant)^54^, and GTH pseudopotentials^55^ for core electrons are used for all calculations. The van der Waals (VDW) interaction was accounted for by employing Grimme’s DFT-D3 method^56^. SCF convergence criterion was set at 10^−6^ for all calculations.

Initial geometries of CsPbX_3_ (X = Cl, Br) nanocrystals were obtained by cutting small cubes (~2.4 nm) from the bulk, exposing the CsX layer at the surface and maintaining overall charge neutrality of the particle^57^. All structures were then optimized in vacuum using the BFGS optimizer imposing non-periodic boundary conditions with a wavelet Poisson solver^58^, setting a maximum force of 5 meV·Å^−1^ (10^–4^ hartree/bohr) as convergence criteria. For the obtained cartesian coordinates, see Supplementary Data 1. For these non-periodic systems, axial strain was simulated by fixing the length of one side of the cube. If the relaxed cubic nanocrystal has side length *a × b × c*, and stress is to be applied along the *z*-direction, “*c*” is fixed at some *cʹ* by constraining the *z* coordinates of both the top and bottom surface-atoms along the *z*-direction, with all other coordinates of all atoms relaxed. % Strain is reported as (*cʹ* − *c*)/*c* × 100%. For calculations involving dimers and tetramers, 2/4 monomers were explicitly considered, but periodic boundary condition was imposed with at least 10 Å vacuum above the surface of the nanocluster to avoid spurious interaction with its periodic image.

## Supplementary information

Supplementary Information

Description of Additional Supplementary Files

Supplementary Data 1

## Data Availability

The X-ray and optical data that support the findings of this study are available in Zenodo.org at https://zenodo.org/record/5607366^59^.
