# Supplementary material for: Spatially resolved fluorescence of caesium lead halide perovskite supercrystals reveals quasi-atomic behavior of nanocrystals
Source: Nat Commun. 2022 Feb 16;13:892. doi: 10.1038/s41467-022-28486-3 (PMC8850480; doi:10.1038/s41467-022-28486-3)
Supplement: Supplementary file 1 — Supplementary Information [file 41467_2022_28486_MOESM1_ESM.pdf]

Supplementary Information

Spatially resolved fluorescence of caesium lead halide perovskite supercrystals reveals quasi-atomic behavior of nanocrystals

Dmitry Lapkin[1,*], Christopher Kirsch[2,*], Jonas Hiller[2,*], Denis Andrienko[3], Dameli Assalauova[1], Kai Braun[2], Jerome Carnis[1], Young Yong Kim[1], Mukunda Mandal[3], Andre Maier[2,4], Alfred J. Meixner[2,4], Nastasia Mukharamova[1], Marcus Scheele[2,4,+], Frank Schreiber[4,5], Michael Sprung[1], Jan Wahl[2], Sophia Westendorf[2], Ivan A. Zaluzhnyy[5], Ivan A. Vartanyants[1,6,+]

1. *Deutsches Elektronen-Synchrotron DESY, Notkestraße 85, D-22607 Hamburg, Germany*

2. *Institut für Physikalische und Theoretische Chemie, Universität Tübingen, Auf der Morgenstelle 18, D-72076 Tübingen, Germany*

3. *Max Planck Institute for Polymer Research, Ackermannweg 10, D-55128 Mainz, Germany*

4. *Center for Light-Matter Interaction, Sensors & Analytics LISA+, Universität Tübingen, Auf der Morgenstelle 15, D-72076 Tübingen, Germany*

5. *Institut für Angewandte Physik, Universität Tübingen, Auf der Morgenstelle 10, D-72076 Tübingen, Germany*

6. *National Research Nuclear University MEPhI (Moscow Engineering Physics Institute), Kashirskoe shosse 31, 115409 Moscow, Russia*

* These authors contributed equally

+To whom correspondence should be addressed

Supplementary Note 1 . Optical absorption and fluorescence of $CsPbBr_2Cl$ and $CsPbBr_3$ nanocrystals in solution

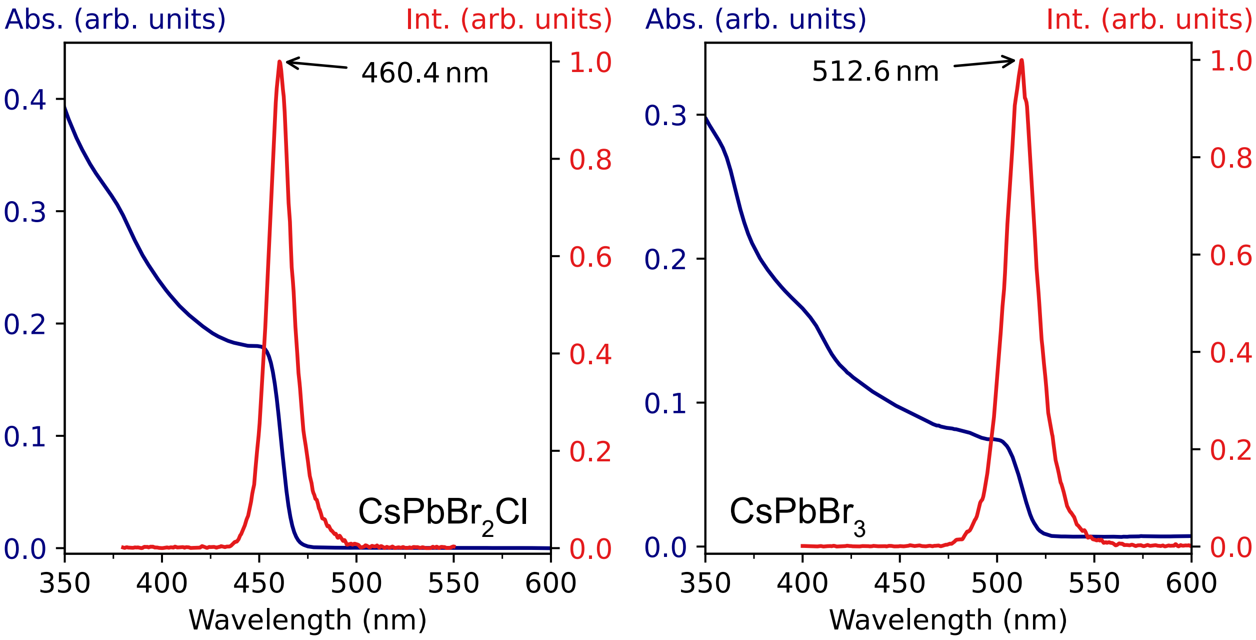

Supplementary Fig. 1. (a) Absorption and emission spectrum of $CsPbBr_2Cl$ NPs dispersed in Toluene. (b) Absorption and emission spectrum of $CsPbBr_3$ NPs dispersed in Hexane.

Optical measurements were performed on a UV-vis-NIR spectrometer (Agilent Technologies, Cary 5000) and a fluorescence spectrometer (PerkinElmer FL8500). All spectra were acquired under ambient conditions in Toluol or Hexane at room temperature (25 °C) in a cuvette of 1 cm pathlength.

Supplementary Note 2. Fluorescence lifetime imaging

A fast fluorescence lifetime imaging microscopy (fast FLIM) image is recorded by scanning the excitation laser over an area of interest while recording the time-resolved fluorescence in the form of a time-correlated single photon counting (TCSPC) histogram at each pixel. The fast lifetime hereby obtained for each pixel is to be understood as the mean photon arrival time after the excitation laser pulse and therefore includes the time the light takes to travel through the instrument. The histogram of the excitation pulse is called the instrument response function (IRF) and was directly measured by the means of scattered light from a clean glass substrate. The physical decay of the sample is obtained by fitting the experimental decay curves recorded at each pixel by employing the IRF in an n-exponential reconvolution with a maximum likelihood estimation method employed for fit optimization.

For both compositions of the investigated self-assembled supercrystals, good results were obtained by fitting a monoexponential reconvolution to the experimental decay curves. Exemplary fits for the center pixels in the FLIM images of the self-assembled supercrystals composed of $CsPbBr_2Cl$ NCs and $CsPbBr_3$ NCs are depicted in Supplementary Fig. 2 and Supplementary Fig. 3 respectively.

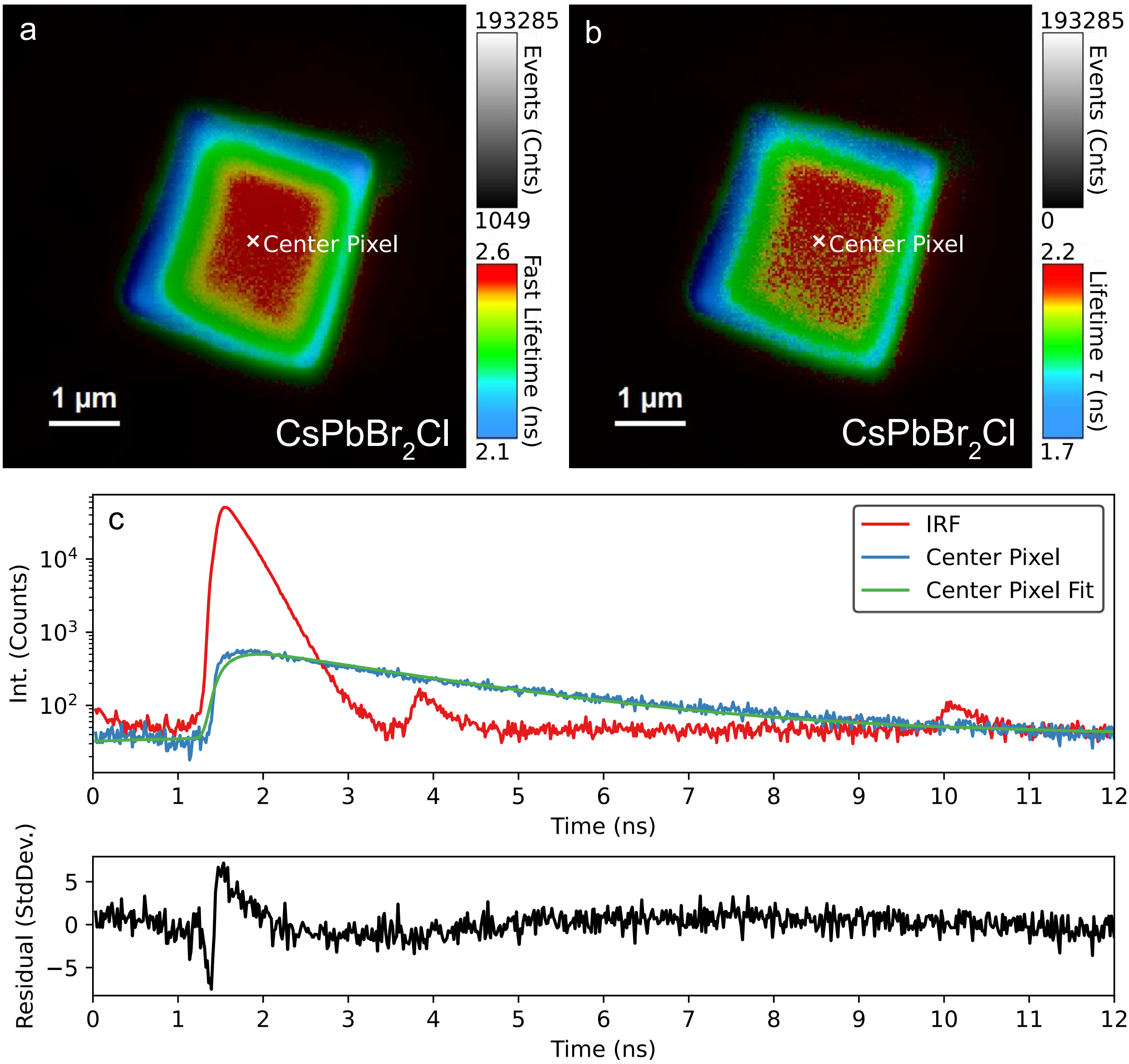

Supplementary Fig. 2. For each pixel of the FLIM images the fluorescence intensity is encoded on a brightness scale while fast lifetimes and fluorescence lifetimes are displayed in RGB false color. (a) Fast FLIM image of a self-assembled $CsPbBr_2Cl$ supercrystal and (b) the corresponding fitted FLIM image obtained through a pixel-by-pixel monoexponential reconvolution. (c) The experimentally acquired IRF as well as an exemplary decay curve of a pixel in the center of the supercrystal and its corresponding monoexponential fit.

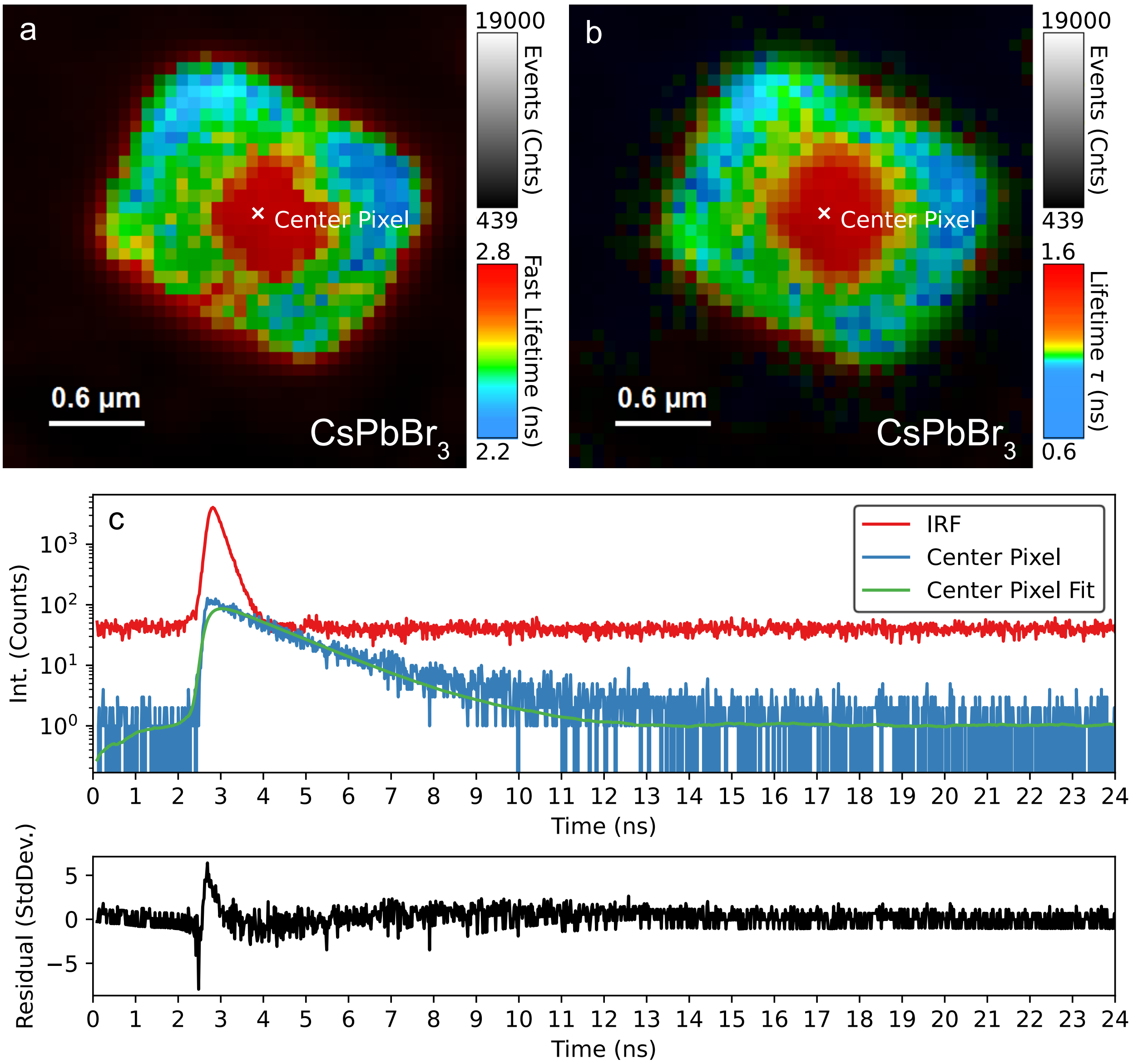

Supplementary Fig. 3. For each pixel of the FLIM images the fluorescence intensity is encoded on a brightness scale while fast lifetimes and fluorescence lifetimes are displayed in RGB false color. (a) Fast FLIM image of a self-assembled $CsPbBr_3$ supercrystal and (b) the corresponding fitted FLIM image obtained through a pixel by pixel monoexponential reconvolution. (c) The experimentally acquired IRF as well as an exemplary decay curve of a pixel in the center of the supercrystal and its corresponding monoexponential fit.

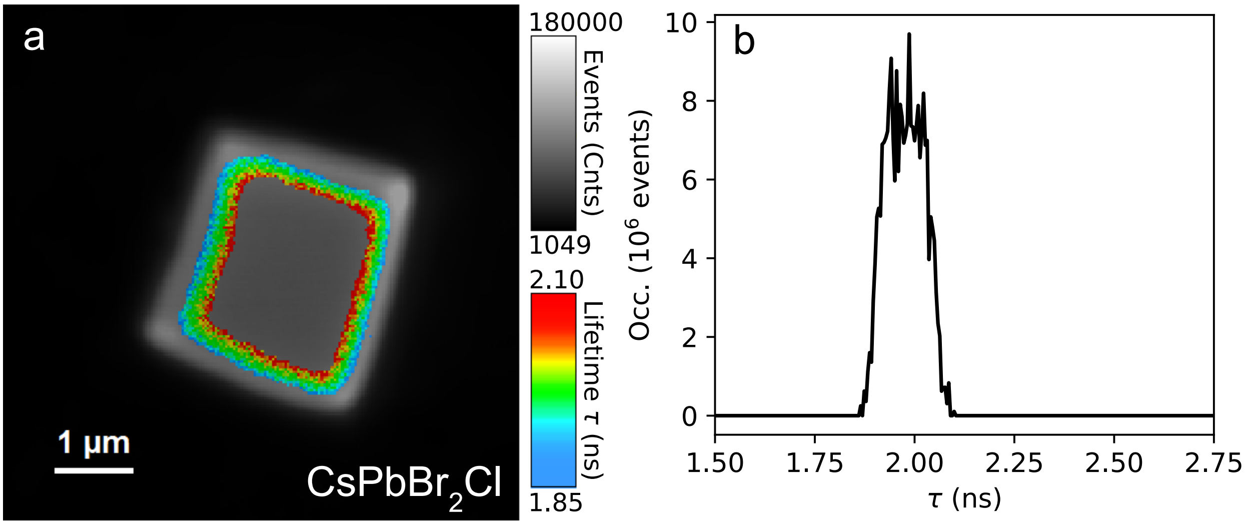

Supplementary Fig. 4. (a) Region of interest (ROI) FLIM image of the intermediate area of the $CsPbBr_2Cl$ supercrystal depicted in Supplementary Fig. 2. (b) The occurrence of the τ-values associated with the monoexponential decay throughout the ROI FLIM image. This ROI FLIM image demonstrates that the decrease of the τ-values when scanning from the center of the supercrystal towards its edges is rather gradual. The "step-like" decrease that Supplementary Fig. 2 seems to imply is due to the chosen RGB color-scaling.

Supplementary Note 2.1. Determining the shortest euclidean distance to the supercrystal edge for each pixel

The first step in the analysis is differentiating between the pixels located inside and those located outside of the supercrystal. This was achieved by assigning the lifetime value 0 to each pixel for which the measured fluorescence intensity is less than half the maximum recorded fluorescence intensity. Because the transition between the inside and the outside of the supercrystal is characterized by a steep drop in the recorded fluorescence intensity, all pixels inside the supercrystal are unaffected and keep their non-zero lifetime value, while all pixels outside of the supercrystal are assigned the lifetime value 0. The masking process for the $CsPbBr_2Cl$ supercrystal is depicted in Supplementary Fig. 5. The pixel-to-edge distance for all pixels inside the supercrystal is then the shortest distance between a pixel with a non-zero lifetime value and a pixel

with the associated lifetime value 0. The free Python machine learning library Scikit-learn was employed for the actual calculations.

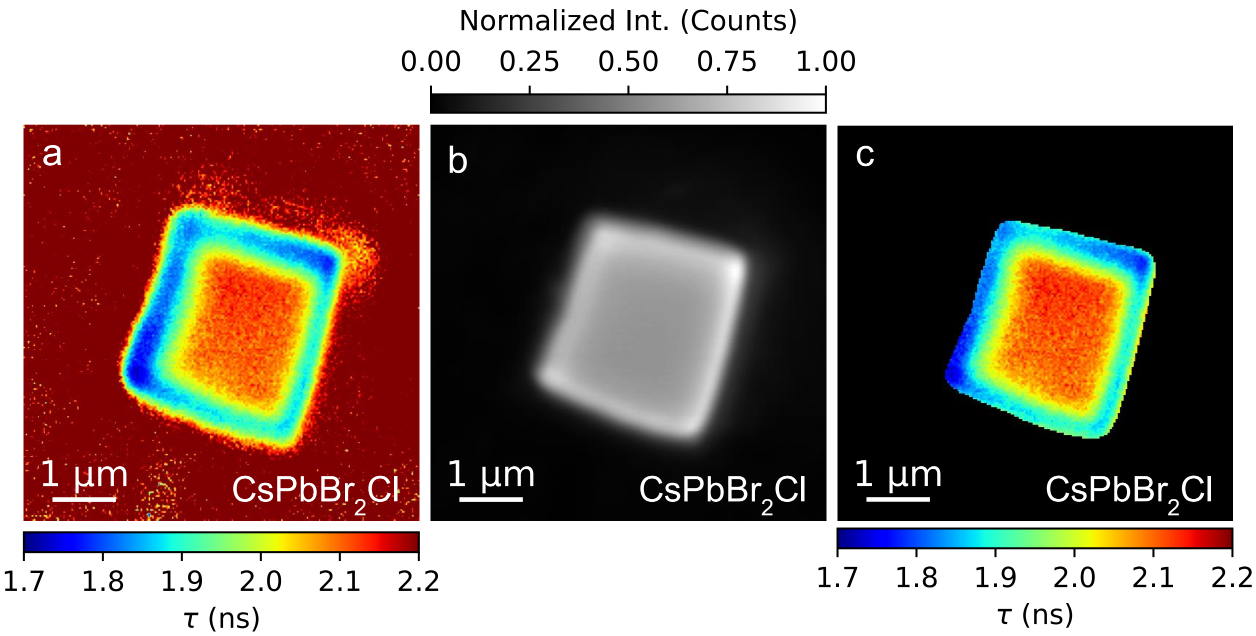

Supplementary Fig. 5. For each of the 200x200 pixels the fluorescence lifetime (a) as well as the fluorescence intensity (b) are recorded. (c) Result of assigning the lifetime value 0 to all pixels for which the recorded fluorescence intensity is less than half the maximum recorded fluorescence intensity. All pixels inside the supercrystal remain unaffected and keep their non-zero lifetime value as determined by the measurement.

Supplementary Note 2.2. Concerning the influence of reabsorption / photon recycling

The reabsorption and subsequent reemission of photons is discussed as a factor contributing to the commonly observed redshifting of the photoluminescence of perovskite nanocrystals when assembled into supercrystals.[1,2] One could reasonably speculate that the effect of such a material geometry dependent photon recycling process is not constant throughout the supercrystal, but that the thinning toward the edges of a not entirely homogenous supercrystal results in an outwardly decreasing influence of reabsorption. It stands to reason that areas with higher rates of reabsorption exhibit lower emission intensity, red-shifted photoluminescence and an increased PL lifetime.

If the spatially resolved optical response of the investigated supercrystals were dominated by changes in the photon propagation due to the supercrystal geometry, we would expect an increase of the PL intensity towards the edges, which would be anticorrelated with a decreasing PL lifetime. The non-normalized single spectra depicted in Supplementary Fig. 6a+b and Supplementary Fig. 7a+b for a $CsPbBr_3$ and a $CsPbBr_2Cl$ supercystal respectively, show that for a typical supercrystal the PL decreases when scanning towards its edges. Furthermore, Supplementary Fig. 6f and Supplementary Fig. 7f show that there is no clear correlation between the PL intensity and lifetime measured at each pixel for both superlattice compositions. While likely playing an important role in the photophysics of supercrystals, the spatially resolved optical data presented here does not show effects that we would associate with changes in the overall reabsorption.

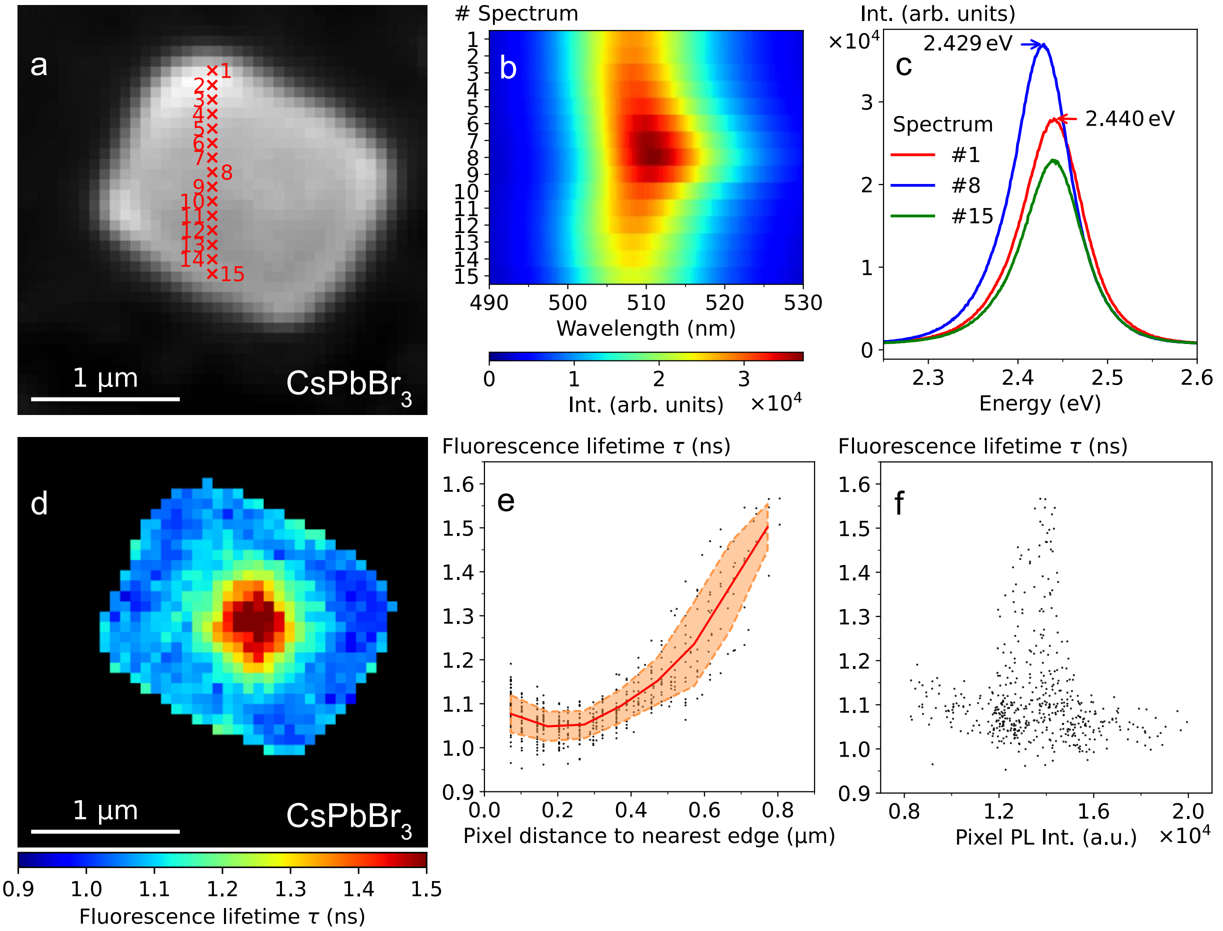

Supplementary Fig. 6: (a) Positions of photoluminescence single spectra, acquired on a $CsPbBr_3$ supercrystal. (b) The corresponding spectra. (c) Selected spectra, acquired at the edges and the center of the supercrystal. (d) Fluorescence lifetime image of a $CsPbBr_3$ NC supercrystal obtained by fitting the experimental time-resolved fluorescence with a monoexponential decay function. (e) Fluorescence lifetime values obtained at each pixel inside the supercrystal as a function of the distance to the nearest edge, where the red line shows the mean value, and the dashed lines indicate the confidence interval of $\pm\sigma$. (f) Fluorescence lifetime values obtained at each pixel inside the supercrystal as a function of the corresponding pixels PL intensity.

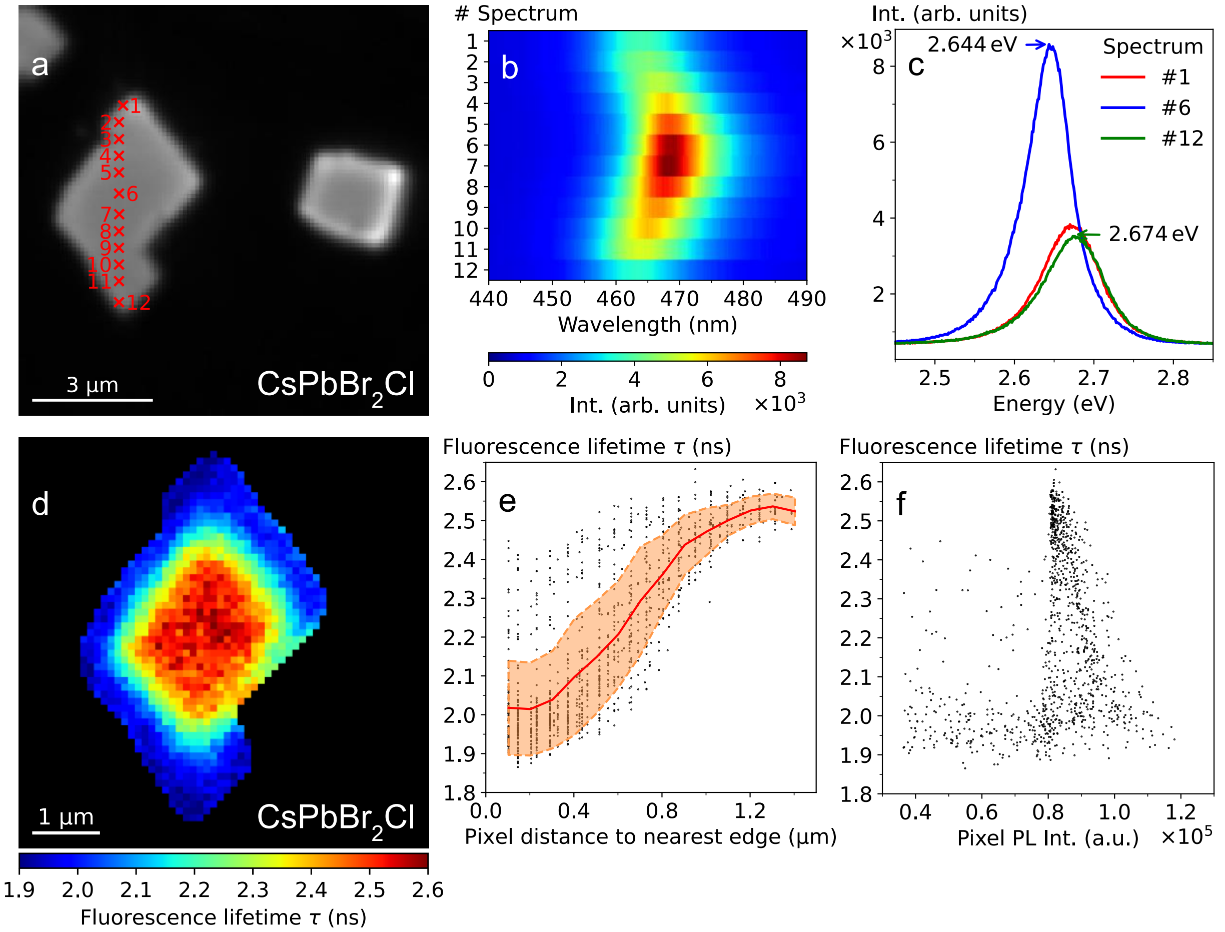

Supplementary Fig. 7: (a) Positions of photoluminescence single spectra, acquired on a $CsPbBr_2Cl$ supercrystal. (b) The corresponding spectra. (c) Selected spectra, acquired at the edges and the center of the supercrystal. (d) Fluorescence lifetime image of a $CsPbBr_2Cl$ NC supercrystal obtained by fitting the experimental time-resolved fluorescence with a monoexponential decay function. (e) Fluorescence lifetime values obtained at each pixel inside the supercrystal as a function of the distance to the nearest edge, where the red line shows the mean value, and the dashed lines indicate the confidence interval of $\pm\sigma$. (f) Fluorescence lifetime values obtained at each pixel inside the supercrystal as a function of the corresponding pixels PL intensity.

Supplementary Note 3. Electron microscopy of $CsPbBr_2Cl$ supercrystals

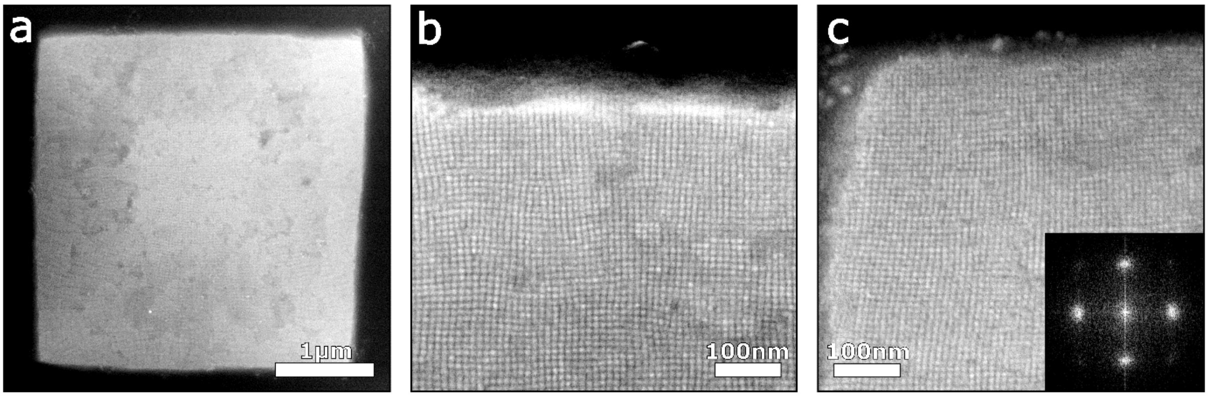

Supplementary Fig. 8: (a) SEM micrograph of self-assembled supercrystals of $CsPbBr_2Cl$ NCs. The NC diameter is rather uniform over the whole crystal (7.3±0.4 nm), as indicated by the high-resolution micrograph of an edge (b) and a corner (c). The inset of (c) corresponds to the FFT of the corresponding micrograph, indicating a homogeneous four-fold symmetry of the NC arrangement.

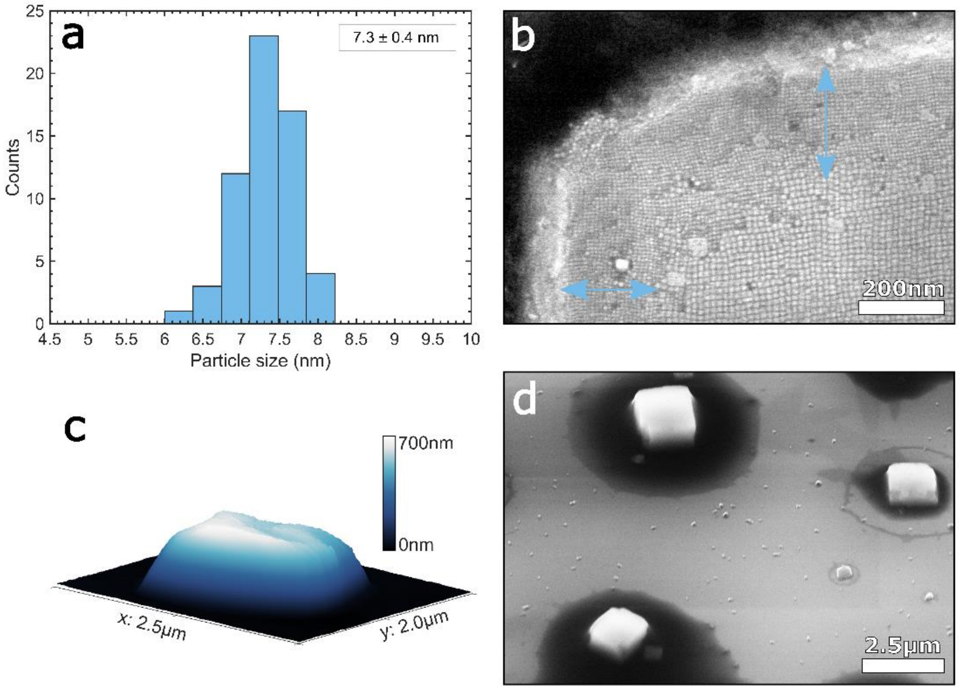

Supplementary Fig. 9: (a) Distribution of the diameter of the $CsPbBr_2Cl$ NCs, measured by SEM. The mean value is 7.3±0.4 nm (size distribution of ~5%). (b) SEM micrograph of the corner of a less faceted supercrystals occasionally featuring NCs of smaller size. The spatial extent of this subpopulation is limited to ~200 nm from the edges, indicated by the blue arrows. (c) 3D AFM map of a supercrystal on a Kapton membrane. (d) SEM micrograph of three supercrystals on a

Si/SiOx wafer under a view angle of 45°. Typical supercrystal thicknesses of 580±120 nm can be observed.

Supplementary Note 4. Atomic structure of the constituting $CsPbBr_2Cl$ NCs

The azimuthally averaged WAXS intensity calculated for the mean pattern for a supercrystal is shown in Supplementary Fig. 10. There are three prominent peaks originating from the atomic lattice of the $CsPbBr_2Cl$ NCs. The peaks can be attributed to 100_{AL}, 110_{AL} and 200_{AL} reflections of a cubic superlattice. The peaks at $q \sim 24.5$ nm^{-1} can be attributed to 210_{AL} reflection which is only partially covered by the detector. The structured background in the range of q = 17-20 nm^{-1} is from the Kapton film. It cannot be fully subtracted due to the anisotropy of scattering from the Kapton film in different spatial point of the scanned area. The peaks were simultaneously fitted by three Gaussian functions:

$$I(q) = \sum_{i=1}^{3} \frac{I_i}{\sqrt{2\pi\sigma_i^2}} \exp\left[-\frac{(q-q_i)^2}{2\sigma_i^2}\right], \qquad \text{(Eq. S1)}$$

where I_i is the integrated intensity, q_i is the momentum transfer and $w_i = 2\sqrt{2\ln 2}\sigma_i$ is the FWHM of the i-th Bragg peak. The peaks are at q_{100} = 10.930±0.005 nm^{-1}, q_{110} = 15.442±0.002 nm^{-1} and q_{200} = 21.900±0.003 nm^{-1} giving the unit cell parameter a_{AL} = 0.574±0.001 nm. The errorbars are the fitting errors. One should note that the expected atomic lattice structure for cesium lead halide perovskites at room temperature is orthorhombic.[3] However, the deviation of the unit cell parameters of such orthorhombic lattice from a cubic lattice is < 2%. We are not able to resolve the peaks of the same order with so small separation due to the size-dependent Scherrer broadening of the Bragg peaks. Thus, we used the pseudocubic indexing of the Bragg peaks, where the 100_{AL} index corresponds to 110_{AL} and 002_{AL} reflections of the orthorhombic structure, the 110_{AL} – to 200_{AL} and 112_{AL}, the 200_{AL} – to 220_{AL} and 004_{AL}.

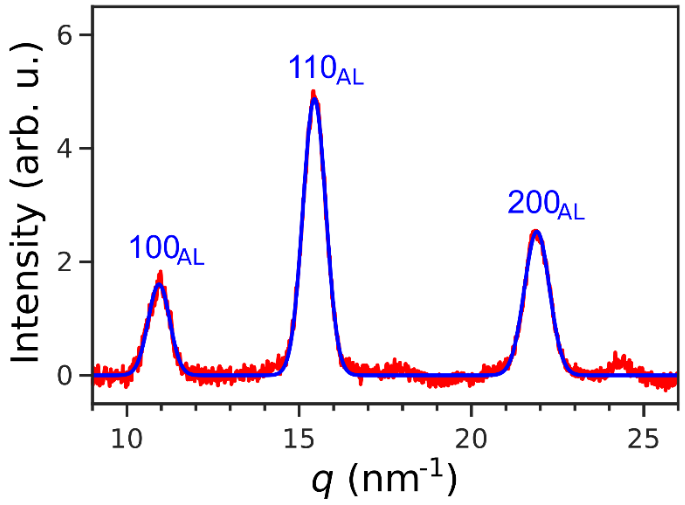

Supplementary Fig. 10. Azimuthally averaged intensity profile in WAXS region (red line) and Gaussian fitting including three peaks (blue line). The peaks are indexed according to a pseudocubic structure.

The FWHMs of the peaks extracted by the fitting are w_{100} = 0.736±0.011 nm^{-1}, w_{110} = 0.784±0.004 nm^{-1}, w_{200} = 0.856±0.08 nm^{-1}. We analyzed the FWHMs of the peaks by the Williamson-Hall method.[4] According to the method, the FWHMs of the Bragg peaks are defined by two factors: the size of coherently scattering domain L and the lattice distortion g (the ratio $\delta a_{\mathrm{AL}}/a_{\mathrm{AL}}$ of the FWHM δa_{AL} of the unit cell parameter distribution around the mean value a_{AL}). If we assume that the coherently scattering domain is a NC, the Williamson-Hall equation can be written as follows:

$$w^2(q) = \left(\frac{2\pi K}{L}\right)^2 + (gq)^2, \qquad \text{(Eq. S2)}$$

where $w(q)$ is the FWHM of the Bragg peak at momentum transfer q, K – a dimensionless shape factor, L – the NC size and g – the lattice distortion.

The first term is the pure Scherrer broadening, where the shape factor K is about 0.85 for the reflections of low orders for a cubic crystallite.[5] One should note, the size of coherently scattering domain can be bigger than a single NC if there are perfectly aligned NCs scattering coherently to the same direction. However, the fact that the WAXS Bragg peaks are much broader in the

azimuthal direction than in the radial (see the main text for the WAXS pattern), indicate a high degree of angular disorder of the NCs (will be discussed below) leading to low probability of such a scenario.

To extract the NC size L and the lattice distortion g, we fitted the experimentally obtained FWHMs and q-values for the present 100_{AL}, 110_{AL} and 200_{AL} peaks with Eq. S2 as shown in Supplementary Fig. 11. The resulting parameters are $L = 6.8 \pm 0.1$ nm and $g = 2.3 \pm 0.1\%$. The resulting NC size L is even smaller than the size $L_{SEM} = 7.3 \pm 0.4$ nm obtained from SEM measurement that indicates the correctness of the assumption that the coherently scattering domain consist of a single NC. The smaller size can be explained by the lattice twinning inside the NC leading to smaller domains and by the limits of the method.

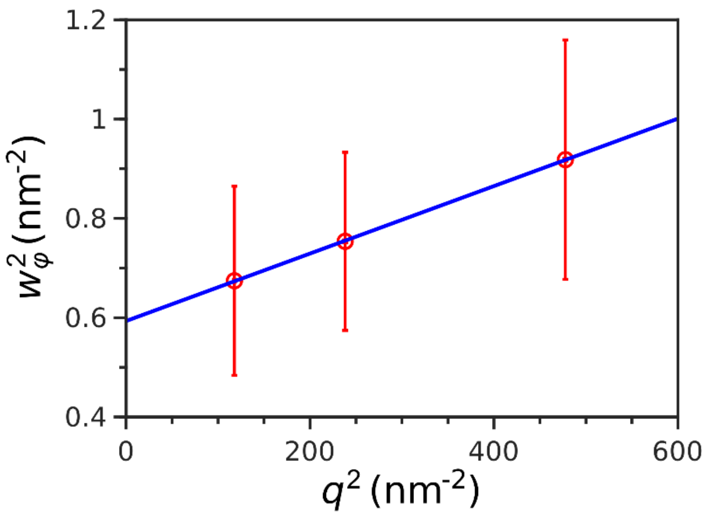

Supplementary Fig. 11. Williamson-Hall plot for the radial FWHM values of the WAXS Bragg peaks. The red points are experimental values, the blue straight line is the best fit.

Supplementary Note 5. Local superlattice structure

The single SAXS diffraction patterns at different spatial points of the supercrystal are quite different from the average diffraction pattern shown in Fig. 3c in the main text. Examples of the single patterns are shown in Supplementary Fig. 12. Clearly, the Bragg peaks do not maintain their positions in both radial and azimuthal directions and change their shape from point to point.

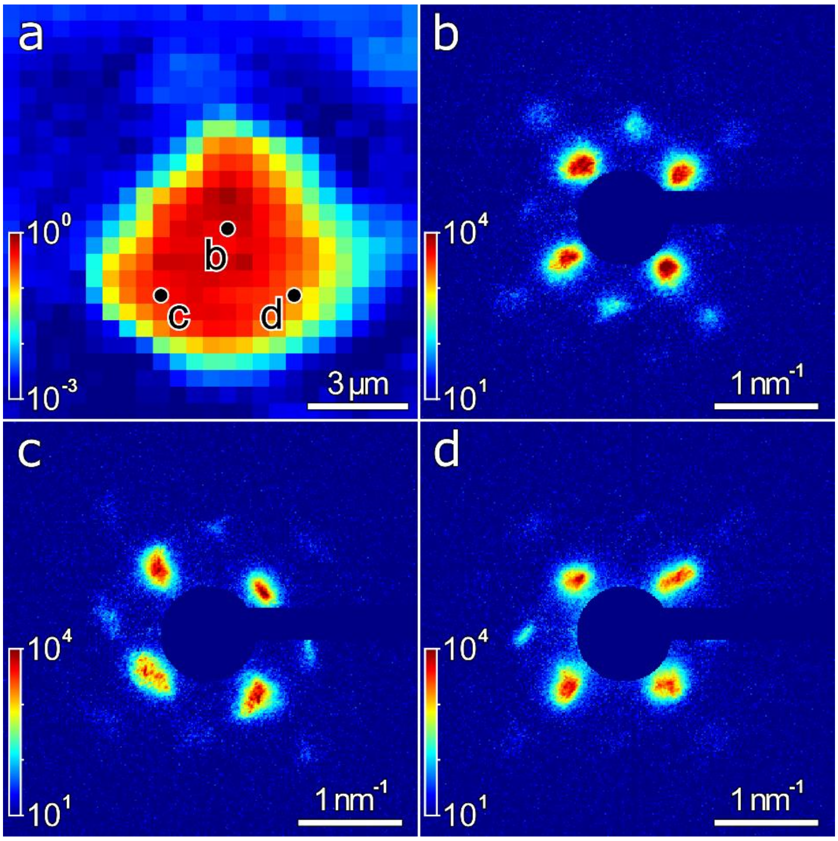

Supplementary Fig. 12. (a) SAXS intensity-based map of the sample. The pixel size is 500 nm. (b) – (d) Examples of single SAXS diffractions patterns collected at the points indicated in panel (a).

To study dependence of their parameters on the spatial position within the sample, we evaluated each single diffraction pattern separately. We fitted each of the first order Bragg peaks in the SAXS region by the Gaussian function:

$$I(q,\varphi) = \frac{I_0}{2\pi\sigma_q\sigma_\omega} \exp\left[-\frac{(q-q_0)^2}{2\sigma_q^2} - \frac{(\omega-\omega_0)^2}{2\sigma_\omega^2}\right], \quad \text{(Eq. S3)}$$

where I_0 is the integrated intensity, q_0 and ω_0 are the radial and azimuthal positions, $w_q = 2\sqrt{2\ln 2}\sigma_q$ and $w_\omega = 2\sqrt{2\ln 2}\sigma_\omega$ are the corresponding FWHMs of the Bragg peak. The fitting was done in the appropriate region of the polar coordinates with single isolated Bragg peak.

There are two Friedel pairs of the Bragg peaks corresponding to reflections from the $(100)_{SL}$ and $(010)_{SL}$ superlattice planes. Counting from the right-pointing horizontal axis, the 1st and 3rd peaks correspond to the $(100)_{SL}$ plane and the 2nd and 4th – to the $(010)_{SL}$ plane. We averaged the intensities, the radial and azimuthal positions and the FWHMs within each pair to get more reliable characteristics of the superlattice planes. The azimuthal coordinates of the 3rd and 4th peaks were corrected by -180° prior to the averaging. Finally, we have two sets of the characteristics defined in Supplementary Fig. 13a. The reciprocal space coordinated were converted into the coordinates of the real-space basis vectors $\mathbf{a}_1$ and $\mathbf{a}_2$.

The azimuthal positions are converted into real space as follows:

$$\varphi_1 = \omega_2 - 90° \quad \text{(Eq. S4)}$$

$$\varphi_2 = \omega_1 + 90° \quad \text{(Eq. S5)}$$

The nearest-neighbor distances a_1 and a_2 are calculated as:

$$a_1 = \frac{2\pi}{q_1 \cdot \sin\gamma} \quad \text{(Eq. S6)}$$

$$a_2 = \frac{2\pi}{q_2 \cdot \sin\gamma} \quad \text{(Eq. S7)}$$

We also used additional azimuthal coordinates that are the azimuthal position of the mean line **M** between the $\mathbf{a}_1$ and $\mathbf{a}_2$:

$$\varphi = \frac{\varphi_1 + \varphi_2}{2} \quad \text{(Eq. S8)}$$

and the angle γ between the real space basis vectors $\mathbf{a}_1$ and $\mathbf{a}_2$, calculated as:

$$\gamma = \varphi_2 - \varphi_1 \quad \text{(Eq. S9)}$$

The definition of the real space coordinates is shown in Supplementary Fig. 13b.

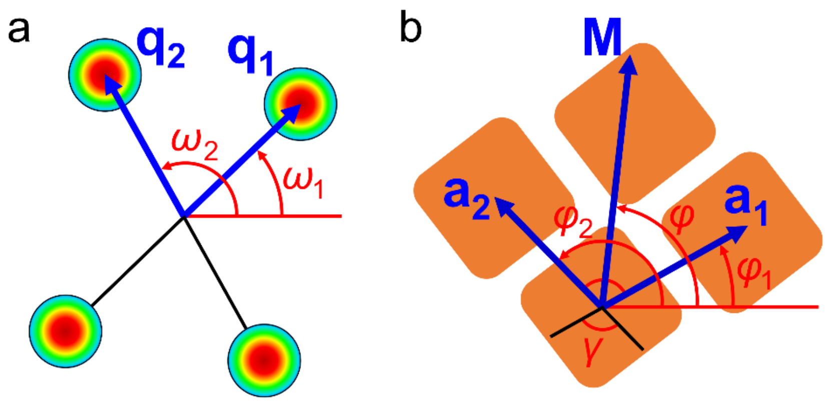

Supplementary Fig. 13. (a) Scheme of the SAXS diffraction pattern from the superlattice. Only the first order Bragg peaks are shown. Two pairs of Bragg peaks are at momentum transfer values q_1 and q_2 and azimuthal positions ω_1 and ω_2, respectively. (b) Scheme of the real space unit cell of the superlattice. The nearest neighbors are at distances a_1 and a_2 with azimuthal positions φ_1 and φ_2, respectively. The mean line **M** between $\mathbf{a_1}$ and $\mathbf{a_2}$ is at azimuthal position φ. The angle between $\mathbf{a_1}$ and $\mathbf{a_2}$ is equal to γ.

The extracted intensities I_1 and I_2 of the Bragg peaks are shown in Supplementary Fig. 14a,d. The scattering areas basically coincide except the upper and right corners. Vanishing of the 1st Bragg peak possibly indicates out-of-plane rotation of the SL on these supercrystal edges as soon as such rotation bring the peak out of the Ewald sphere.

The extracted momentum transfers q_1 and q_2 associated with the superlattice plane spacings are shown in Supplementary Fig. 14b,e. As it is clear from the figure, the momentum transfers grow on the edges of the supercrystal. It indicates contraction of the superlattice that is thoroughly discussed in the main text as well as its anisotropy.

The extracted azimuthal positions ω_1 and ω_2 are shown in Supplementary Fig. 14c,f. They clearly indicate rotation of the superlattice around the incident beam (normal to the substrate). The rotation is also discussed in the main text.

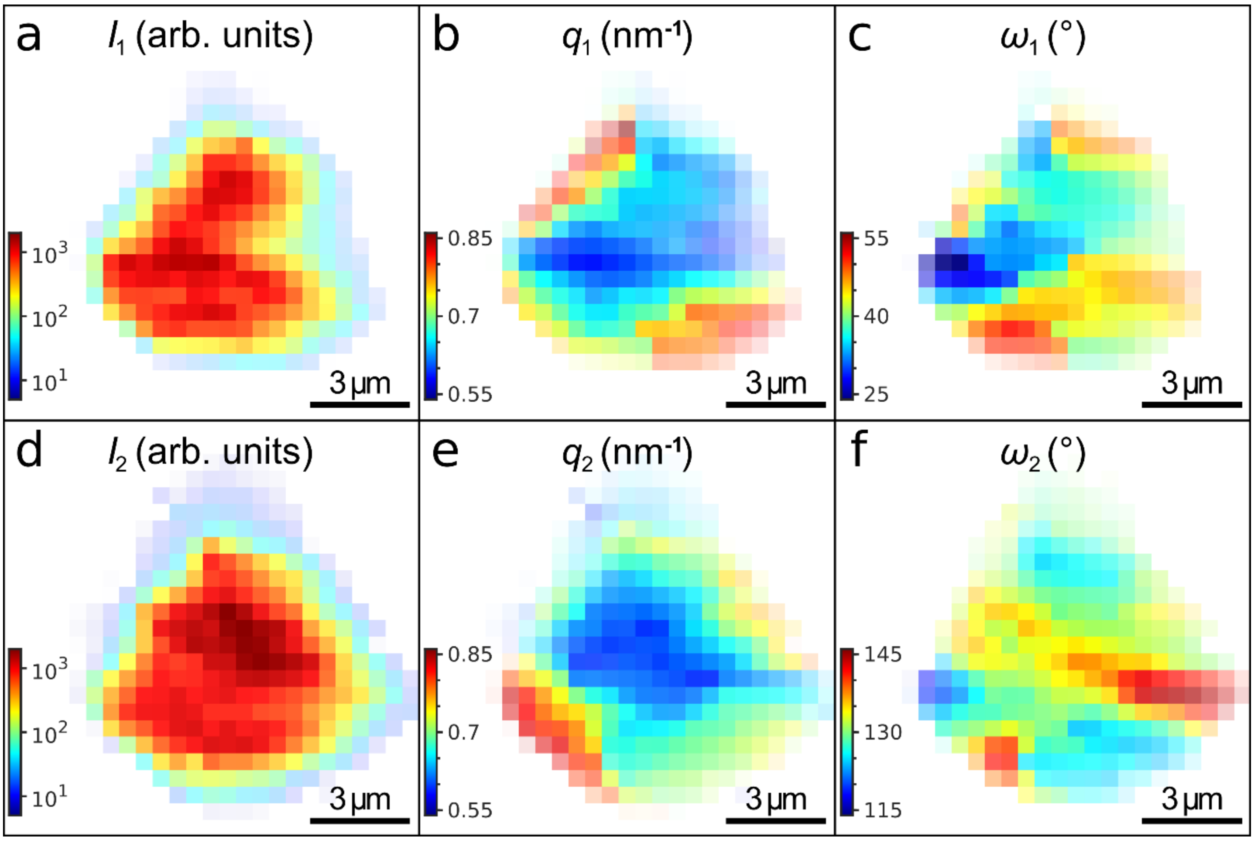

Supplementary Fig. 14. Mean extracted parameters: (a, d) intensities, (b, e) momentum transfer values and (c, f) azimuthal positions of (a – c) the 1st and 3rd and (d – f) the 2nd and 4th Bragg peaks. The azimuthal positions are counted counterclockwise from a horizontal axis pointing to the right. The azimuthal position of the 3rd and 4th were corrected by -180° before averaging with their counterparts. The pixel size is 500 nm.

The extracted FWHMs in the radial direction w_{q1} and w_{q2} are shown in Supplementary Fig. 15a,c. Interestingly, in contrast to the momentum transfer values q_1 and q_2, the main deviations in FWHMs happen on the edges to which the crystallographic axes are perpendicular. This means lower dispersion in the superlattice plane separation normal to the edge of the supercrystal.

The extracted FWHMs in the azimuthal direction $w_{\omega 1}$ and $w_{\omega 2}$ are shown in Supplementary Fig. 15b,d. There is no clear trend in the behavior of these parameters in respect to the spatial position inside the supercrystal. But most of the point having high FWHM values are located in the middle of the supercrystal, indicating higher dispersion of the angle γ between the lattice vectors a_1 and a_2 as well as of the superlattice orientation angle φ.

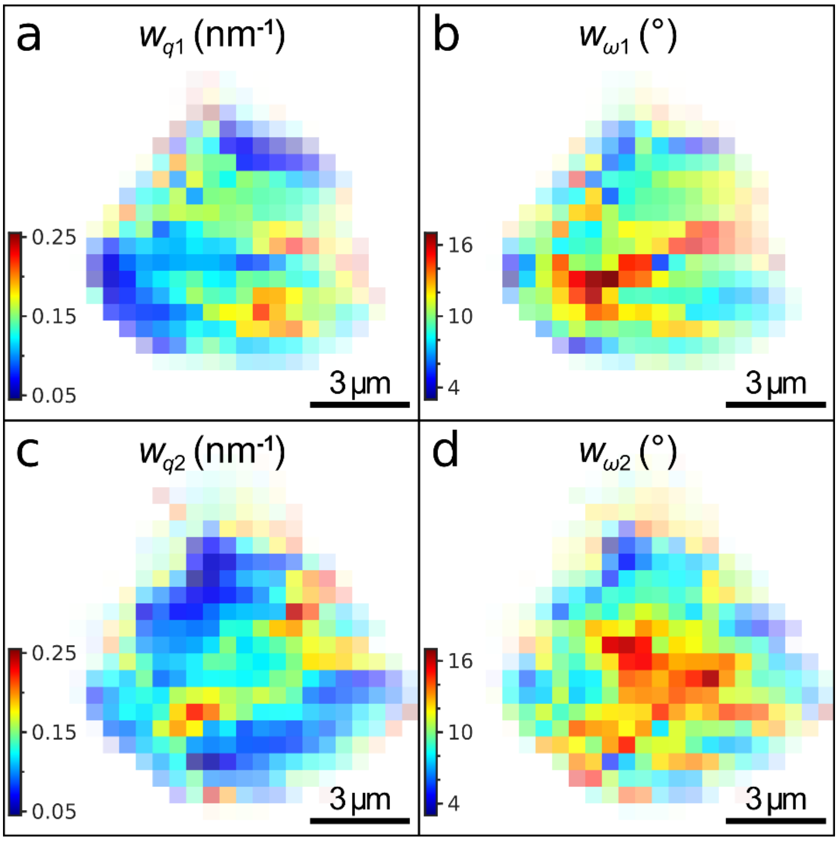

Supplementary Fig. 15. Mean extracted FWHMs in (a, c) radial and (b, d) azimuthal directions of (a,b) the 1st and 3rd and (c,d) the 2nd and 4th Bragg peaks. The pixel size is 500 nm.

The calculated lengths of the lattice vectors a_1 and a_2 are shown in Supplementary Fig. 16. The distance between the adjacent NCs decrease in both directions on the edges of the supercrystal, but the effect is higher in the direction parallel to the nearest supercrystal edge. For example, in the point 1 in Supplementary Fig. 16, the distance along $\mathbf{a}_1$, which is pointing to the top-right parallel to the nearest edge, is smaller than along $\mathbf{a}_2$, which is normal to the nearest edge. On the contrary, in the point 2, where $\mathbf{a}_2$ is parallel to the nearest edge, the distance along this direction is smaller than along $\mathbf{a}_1$. The anisotropy of the lattice shrinkage is better visible on the map of the a_2/a_1 ratio that is shown and discussed in the main text.

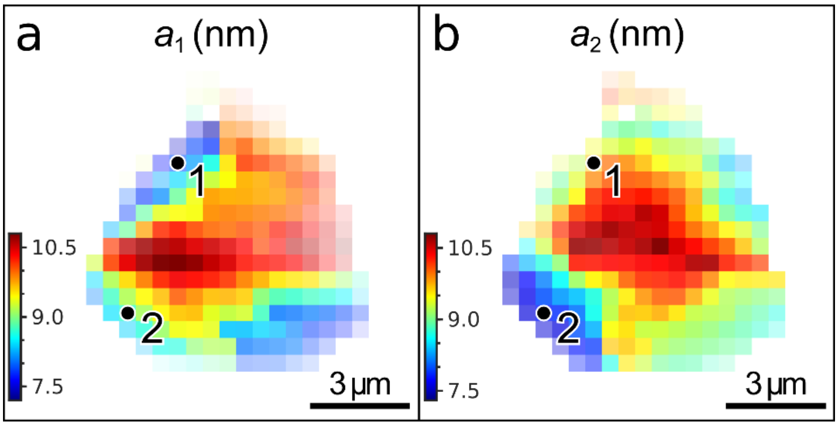

Supplementary Fig. 16. Maps of the calculated lengths of the lattice vectors a_1 and a_2. The points 1 and 2 are discussed in the text. The pixel size is 500 nm.

Supplementary Note 6. NCs orientation inside the SL

In WAXS region, there are four Bragg peaks present on most of the diffraction pattern, as shown on the average one in Supplementary Fig. 17. Analogous to the SAXS analysis, we fitted each of the peaks separately for each spatial point within the sample with the 2D Gaussian functions (Eq. S3).

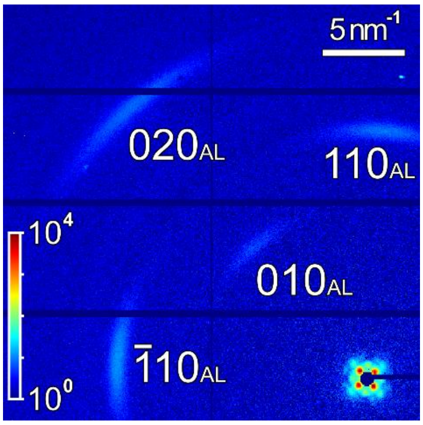

Supplementary Fig. 17. Average WAXS diffraction pattern with four prominent Bragg peaks. The indexes are given for pseudocubic atomic lattice oriented along [001] axis.

The intensities of the Bragg peaks extracted this way are shown in Supplementary Fig. 18. 010_{AL}, 020_{AL}, 110_{AL} and $\bar{1}10_{AL}$ peaks are detected in the spatial points within the supercrystal, though lower intensity of the WAXS reflections did not allow detecting them on the very edges. The 010_{AL} reflection is registered on even smaller area, because it has the lowest intensity among the peaks.

The intensities are not uniform within the supercrystal that can indicate different thickness of the sample or slight out-of-plane rotation of the NCs. The intensities of 010_{AL}, 020_{AL} and 110_{AL} reflections change the same way, while the intensity of $\bar{1}10_{AL}$ reflection stay almost constant. The inhomogeneous thickness would lead to the similar changes in intensities of all reflections, thus, the change most likely is due to the out-of-plane rotation of the NCs. The constant intensity of $\bar{1}10_{AL}$ reflection indicates that the rotation happens around an axis close to the $[\bar{1}10]_{AL}$ one. The

changes in intensity of 010_{AL} and 020_{AL} reflections are qualitatively similar to the changes in intensity I_2 of 010_{SL} reflection (see Supplementary Fig. 14d) that indicates simultaneous rotation of the NCs and SL keeping their mutual orientation. The changes in SAXS intensity are smaller due to lower effect of the Ewald's sphere curvature.

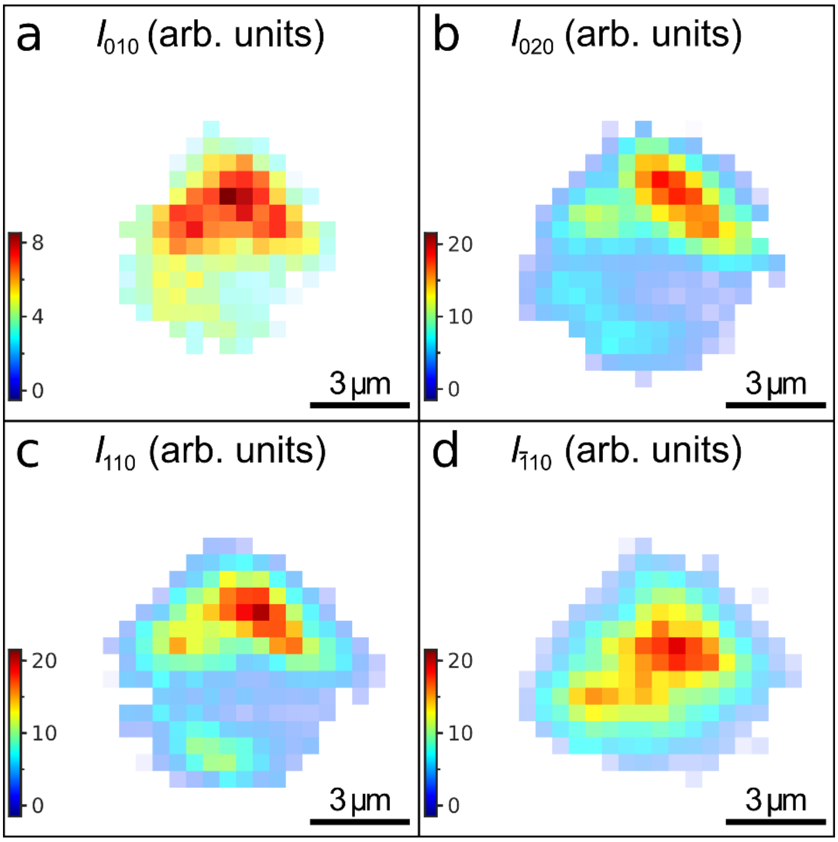

Supplementary Fig. 18. Extracted intensities of the WAXS Bragg peaks: **a**) 010_{AL}, **b**) 020_{AL}, **c**) 110_{AL} and **d**) $\bar{1}10_{AL}$. The pixel size is 500 nm.

The mean extracted momentum transfer values are q_{010} = 10.87±0.05 nm^{-1}, q_{110} = 10.85±0.05 nm^{-1}, $q_{\bar{1}10}$ = 10.91±0.03 nm^{-1}, q_{020} = 21.86±0.04 nm^{-1}, that is in a good agreement with the values obtained from the average radial profiles discussed above in Supplementary Note 5. The errorbars here represent the standard deviation of the values across the sample. The momentum transfer values do not depend on the spatial position on the sample, as shown in Supplementary Fig. 19. The deviation is due to the noise together with the low intensities of the peaks themselves.

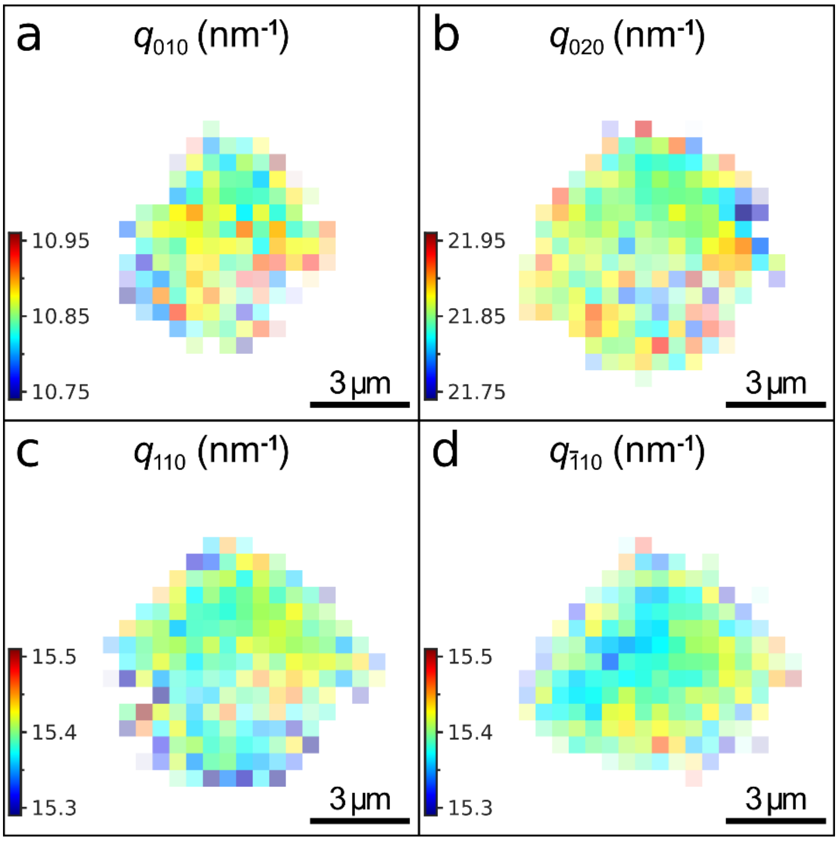

Supplementary Fig. 19 Extracted momentum transfers of the WAXS Bragg peaks: (a) 010_{AL}, (b) 020_{AL}, (c) 110_{AL} and (d) $\bar{1}10_{AL}$. The pixel size is 500 nm.

The calculated from the q-values unit cell parameter is shown in Supplementary Fig. 20a. It does not change within the supercrystal and remains constant at the value of a_{AL} = 0.576±0.002 nm. To better visualize the change in the unit cell parameter, we plot the values for each pixel against the distance of this pixel to the nearest supercrystal edge in Supplementary Fig. 20b.

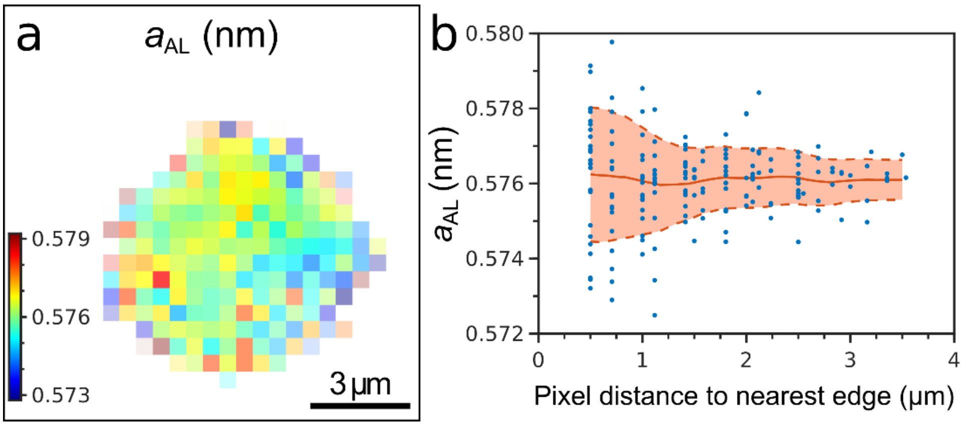

Supplementary Fig. 20. (a) Calculated unit cell parameter a_{AL} of the pseudo-cubic atomic lattice of the NCs and (b) the same value for each pixel against the distance from this pixel to the nearest edge of the supercrystal. The red line shows the mean value, the dashed lines indicate the confidence interval of $\pm\sigma$. The pixel size in (a) is 500 nm.

The FWHMs in radial direction, shown in Supplementary Fig. 21, do not show any correlations with the spatial position on the sample as well. The mean values are $w_{q,010}$ = 0.73±0.10 nm^{-1}, $w_{q,110}$ = 0.75±0.09 nm^{-1}, $w_{q,\bar{1}10}$ = 0.73±0.07 nm^{-1} and $w_{q,020}$ = 0.85±0.11 nm^{-1}, that is in good agreement with the values from the average radial profile discussed above in Supplementary Note 4. The errorbars here represent the standard deviation of the values across the sample.

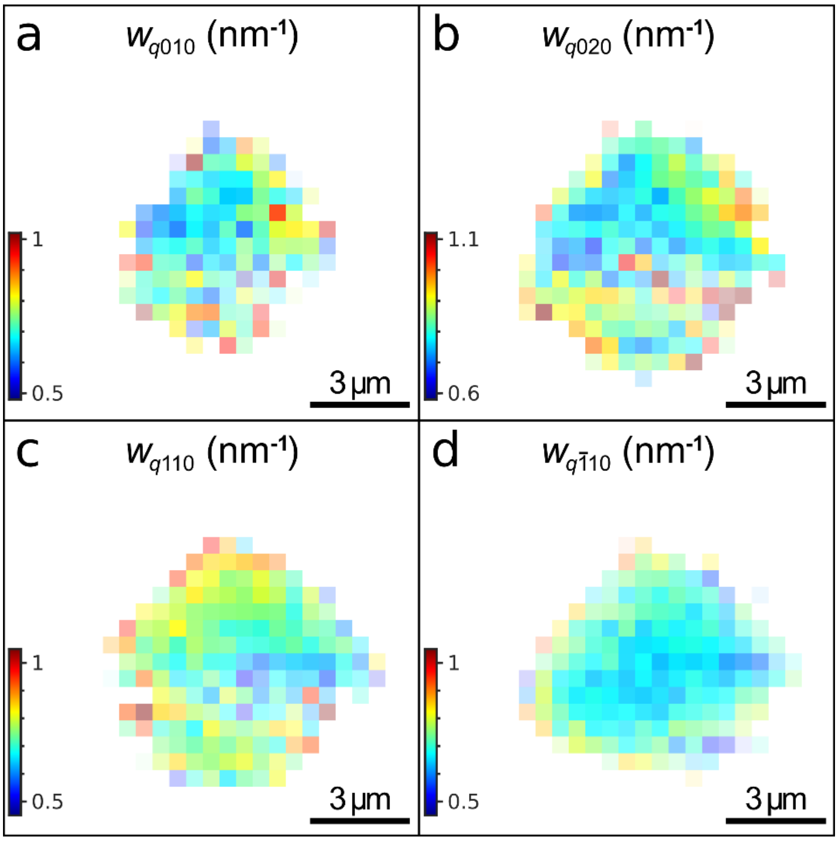

Supplementary Fig. 21. Extracted radial FWHMs of the WAXS Bragg peaks: (a) 010_{AL}, (b) 020_{AL}, (c) 110_{AL} and (d) $\bar{1}10_{AL}$. The pixel size is 500 nm.

We used the radial FWHMs to extract the atomic lattice distortion of the NCs by the Williamson-Hall method (Eq. S2). The lattice distortion g_q (the ratio $\delta a_{AL}/a_{AL}$ of the FWHM δa_{AL} of the unit cell parameter distribution around the mean value a_{AL}) was calculated as follows:

$$g_q = \langle \frac{1}{q_i} \sqrt{(w_i)^2 - \left(\frac{2\pi K}{L}\right)^2} \rangle_i, \qquad \text{(Eq. S10)}$$

where w_i are the radial FWHMs for the present peaks, q_i are the momentum transfer values of the corresponding peaks, K is the shape constant, L is the NCs size and the averaging is performed over all present Bragg peaks. The shape constant K was discussed in Supplementary Note 4; the NCs size was fixed at L = 6.8 nm obtained from the radial profile as described in the same Supplementary Note 4. The resulting values of the atomic lattice distortion are shown in Figure 6 in the main text. The mean value is g_q = 1.5 ± 0.9% that is in good agreement with the values

obtained from the average radial profile discussed in Supplementary Note 1. The distortion gets slightly higher on the edges of the supercrystal that can be explained by the contraction of the NCs together with the superlattice. To better visualize the change in distortion, we plot the values for each pixel against the distance of this pixel to the nearest supercrystal edge in Fig. 6b in the main text. The atomic lattice distortion grows from < 1% in the middle of the supercrystal up to > 2% on the edges. The trend is even more evident for another sample, described in Supplementary Note 7. Here the effect is less pronounced, probably, because of lower intensity of the WAXS Bragg peaks causing higher noise.

Since the NCs have pseudocubic atomic lattice (all angles are equal to 90°), the azimuthal position of the WAXS Bragg peaks directly correspond to the azimuthal orientation of the supercrystal unit cell basis vectors $\mathbf{a_1}$ and $\mathbf{a_2}$. Thus, the azimuthal positions in real space are equal to the azimuthal positions in reciprocal space $\varphi_{hkl} = \omega_{hkl}$. The extracted azimuthal positions for all four peaks are shown in Supplementary Fig. 22. The NCs are clearly rotating in-plane as soon as the positions change for different peaks together from point to point. As expected, the difference between the positions remains constant, e.g. $\varphi_{\bar{1}10} - \varphi_{110} = 90°$, $\varphi_{\bar{1}10} - \varphi_{020} = 45°$ etc. We calculated the average azimuthal NC position ψ collinear to the 010_{AL} vector as $\psi = \langle \varphi_{010}, \varphi_{020}, \varphi_{110} + 45°, \varphi_{\bar{1}10} - 45° \rangle$, where the angle brackets denote averaging over the four angles. The angle ψ is used to study the azimuthal position of the NCs. It is shown in Fig. 5c and discussed in the main text.

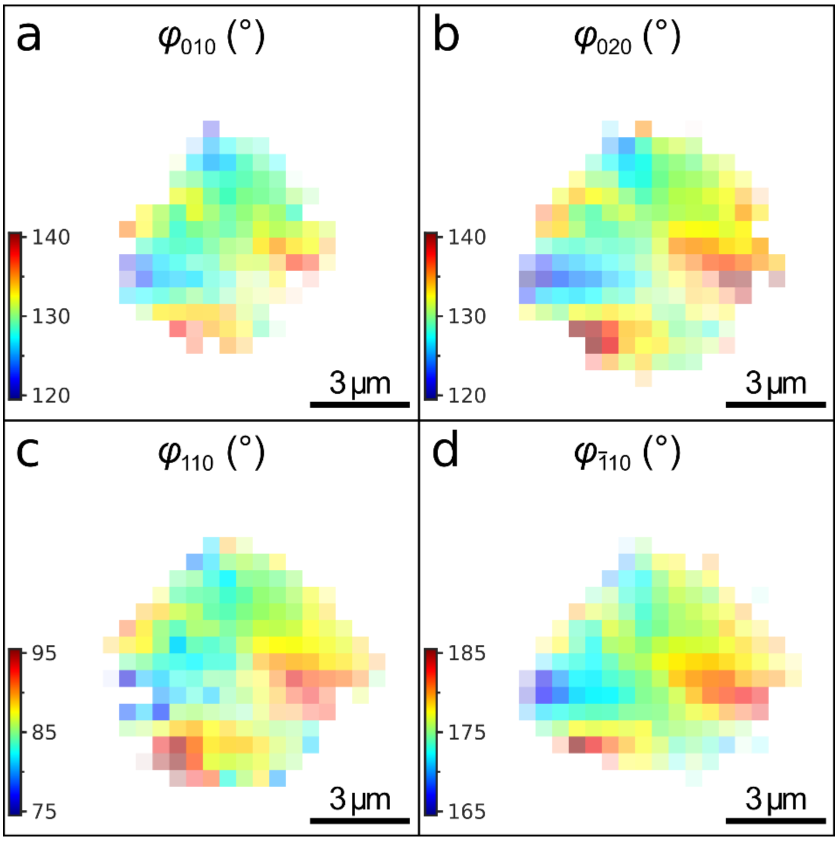

Supplementary Fig. 22. Extracted azimuthal positions of the WAXS Bragg peaks: (a) 010_{AL}, (b) 020_{AL}, (c) 110_{AL} and (d) $\bar{1}10_{AL}$. The positions are counted counterclockwise from a horizontal axis pointing to the right. The pixel size is 500 nm.

We studied the relative azimuthal orientation of the NCs and the SL comparing the azimuthal positions of the lattice vectors obtained from the Bragg peak analysis described above. The differences in angles between the $[010]_{AL}$ axis of the NCs and the mean line **M** between the superlattice vectors $\mathbf{a_1}$ and $\mathbf{a_2}$ $\Delta = \psi - \varphi$; between the $[010]_{AL}$ and $\mathbf{a_1}$ $\Delta_1 = \psi - \varphi_1$; between the $[010]_{AL}$ and $\mathbf{a_2}$ $\Delta_2 = \psi - \varphi_2$ are shown in Supplementary Fig. 23. Clearly, the angle Δ has narrower distribution with the mean value $\langle\Delta\rangle$ =45.2±1.7°. For comparison, the angles Δ_1 *and* Δ_2 have broader distribution with the mean values $\langle\Delta_1\rangle$ = 90.1±3.5° and $\langle\Delta_2\rangle$ = 0.8±3.8°. This is rather expected, because the angles between the NC crystallographic axes are constant, while the angle between the superlattice vectors changes from point to point in relatively broad range, as shown in Fig. 4d and discussed in the main text. At the same time, the angle between the mean lines between the superlattice vectors (e. g. **M** between $\mathbf{a_1}$ and $\mathbf{a_2}$ and **M'** between $\mathbf{a_1}$ and -$\mathbf{a_2}$) is always

equal to 90° and does not depend on the length and orientation of the vectors $\mathbf{a}_1$ and $\mathbf{a}_2$. It makes possible keeping the mutual orientation between the $[100]_{AL}$ and $[010]_{AL}$ axes and the mean lines **M** and **M'**.

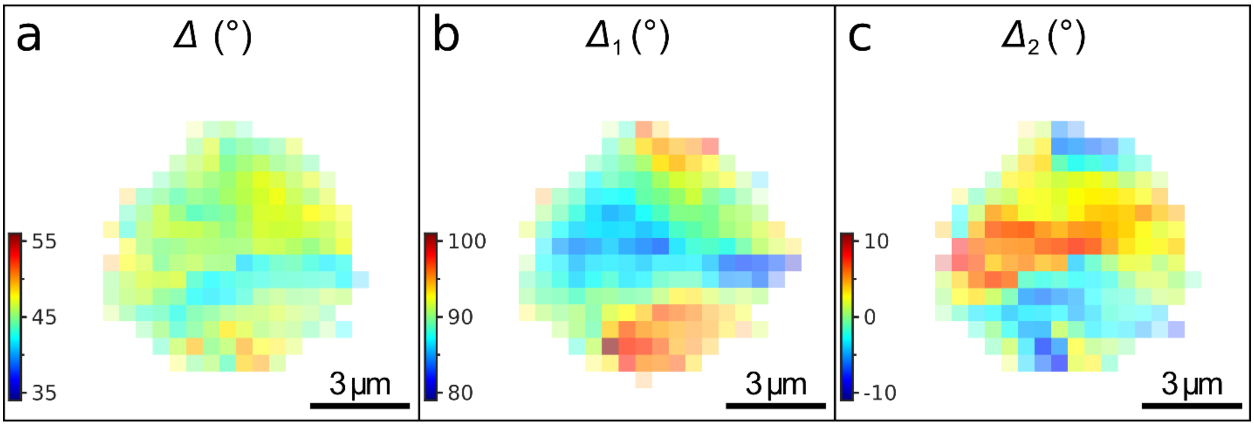

Supplementary Fig. 23. Relative angle between the direct lattice vectors of the NCs and the SL: (a) between $[010]_{AL}$ and the mean line **M** between the $\mathbf{a}_1$ and $\mathbf{a}_2$; (b) between $[010]_{AL}$ and $\mathbf{a}_1$; (c) between $[010]_{AL}$ and $\mathbf{a}_2$. The pixel size is 500 nm.

The azimuthal FWHMs shown in Supplementary Fig. 24 show clear dependence on the spatial position within the supercrystal. The FWHMs grow from 12° in the middle of the supercrystal up to 24° on the edges indicating higher azimuthal disorder of the NCs there.

We used the azimuthal FWHMs to extract the NCs angular disorder by the Williamson-Hall method. In this case, the lattice distortion g from Eq. S2 is the FWHM $\delta\psi$ of angular distribution of the NCs around their mean position given by the azimuthal peak positions. The FWHM $\delta\psi$ was calculated as follows:

$$\delta\psi = \langle \frac{1}{q_i}\sqrt{(w_i q_i)^2 - \left(\frac{2\pi K}{L}\right)^2} \rangle_i, \qquad \text{(Eq. S11)}$$

where w_i are the azimuthal FWHMs (in radians) for the present peaks, q_i are the momentum transfer values of the corresponding peaks, K is the shape constant, L is the NCs size and the averaging is performed over all present Bragg peaks. The shape constant K was discussed in Supplementary Note 4; the NCs size was fixed at L = 6.8 nm obtained from the radial profile as

described in the same Supplementary Note 4. The resulting values of the FWHM are shown in Figure 5d and discussed in the main text.

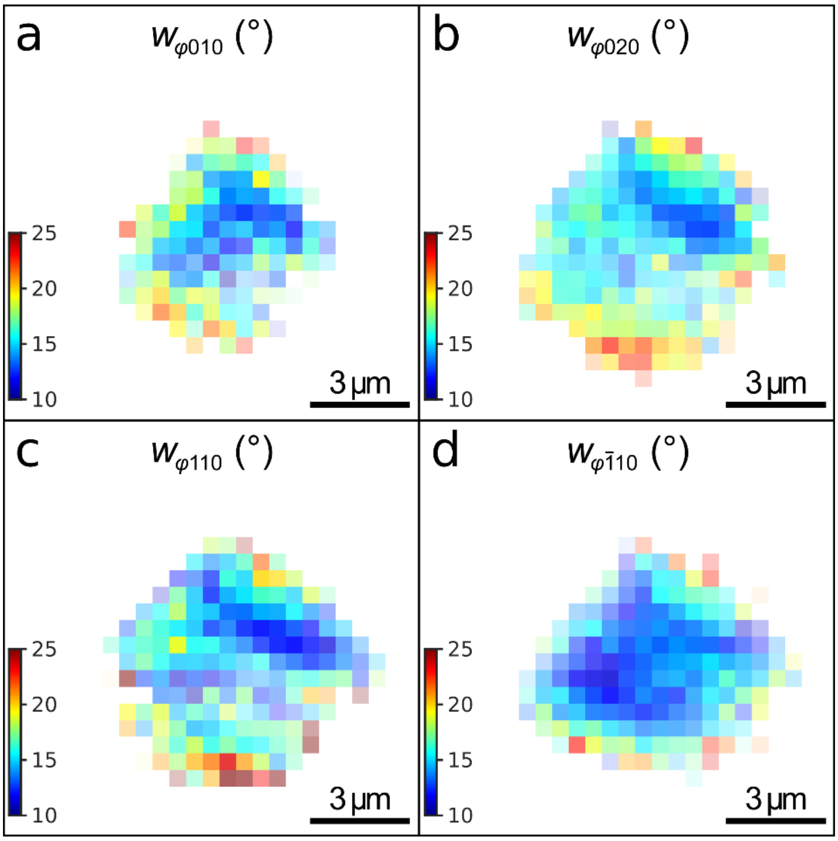

Supplementary Fig. 24. Extracted azimuthal FWHMs of the WAXS Bragg peaks: (a) 010_{AL}, (b) 020_{AL}, (c) 110_{AL} and (d) $\bar{1}10_{AL}$. The pixel size is 500 nm.

Supplementary Note 7. Another example of a supercrystal

All studied supercrystals behave similarly. Here we present the main results obtained for one more supercrystal. The average WAXS and SAXS patterns as well as the SAXS-based diffraction map are shown in Supplementary Fig. 25. The patterns look very similar to the ones observed for the sample described in the main text. The local structure was analyzed the same way as described above. The only difference is the WAXS analysis, performed only for three peaks 010_{AL}, 020_{AL} and $\bar{1}10_{AL}$. The fourth 110_{AL} peak hardly fitted into the detector in this case and was excluded from the consideration.

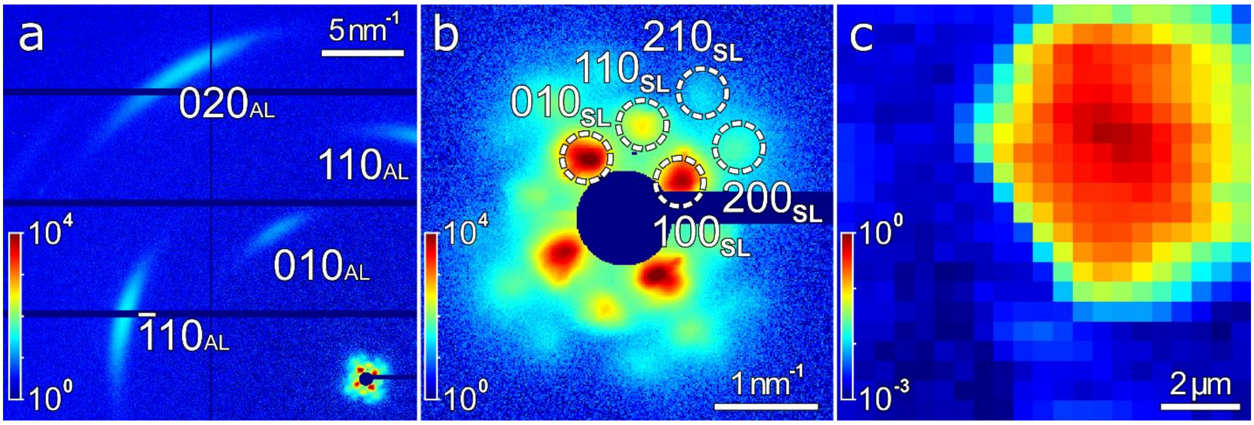

Supplementary Fig. 25. Average WAXS (a) and SAXS (b) patterns and (c) SAXS-based diffraction map of the second sample. The Bragg peaks are indexed according to the "pseudocubic" structure of the atomic lattice and simple cubic structure of the superlattice. The pixel size in c) is 500 nm.

The main extracted parameters of the superlattice are shown in Supplementary Fig. 26. The main observations are the same as for the sample described in the main text. The superlattice anisotropically shrinks on the edges of the supercrystal. The contraction happens preferentially in the directions parallel to the nearest supercrystal edge. The angle between the basis vectors $\mathbf{a}_1$ and $\mathbf{a}_2$ changes in the range of 75-105° and the superlattice rotates in-plane as indicated by the azimuthal position φ of the mean line $\mathbf{M}$ between $\mathbf{a}_1$ and $\mathbf{a}_2$.

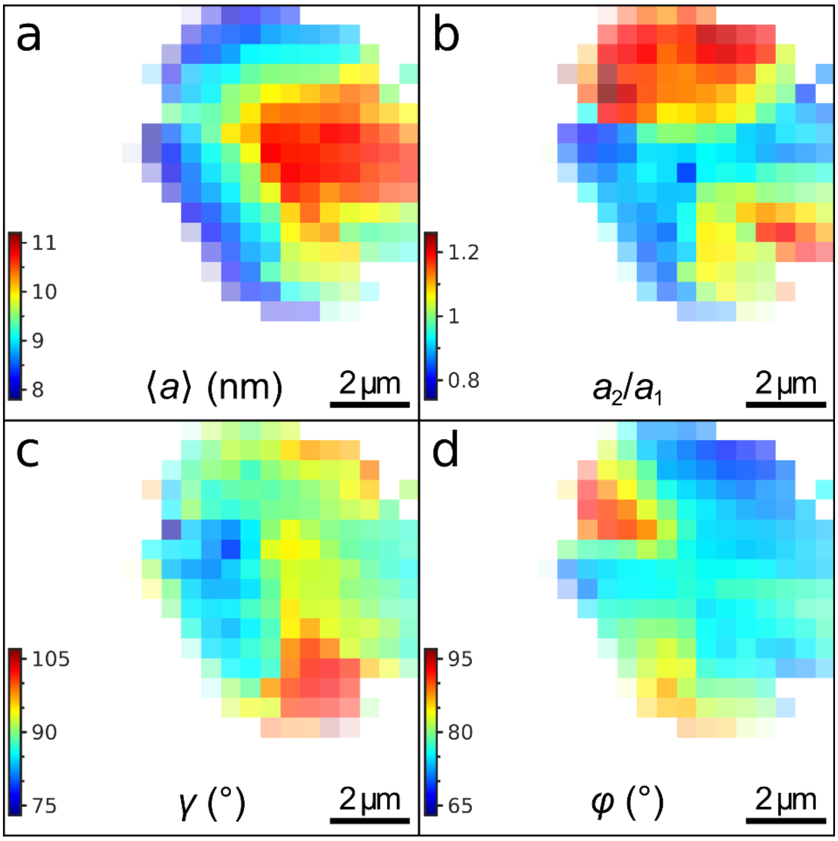

Supplementary Fig. 26. Extracted superlattice parameters: (a) average unit cell parameter $\langle a \rangle = (a_1 + a_2)/2$; (b) ratio a_2/a_1 of the NC spacings along the basis vectors $\mathbf{a}_2$ and $\mathbf{a}_1$; (c) angle γ between the basis vectors $\mathbf{a}_1$ and $\mathbf{a}_2$; (d) azimuthal position φ of the mean line **M** between the basis vectors $\mathbf{a}_1$ and $\mathbf{a}_2$. The pixel size is 500 nm.

The main extracted parameters of the atomic lattice are shown in Supplementary Fig. 27. The main difference with the sample described in the main text is the homogeneous intensity of the WAXS Bragg peaks decreasing on the edges. It indicates absence of the out-of-plane rotations of the NCs. But the NCs rotate in-plane correlated with the mean line **M** between the $\mathbf{a}_1$ and $\mathbf{a}_2$ superlattice vectors as can be seen from the difference angle $\Delta = \psi - \varphi$. The angular disorder of the NCs grows on the edges the same way as for the sample described in the main text.

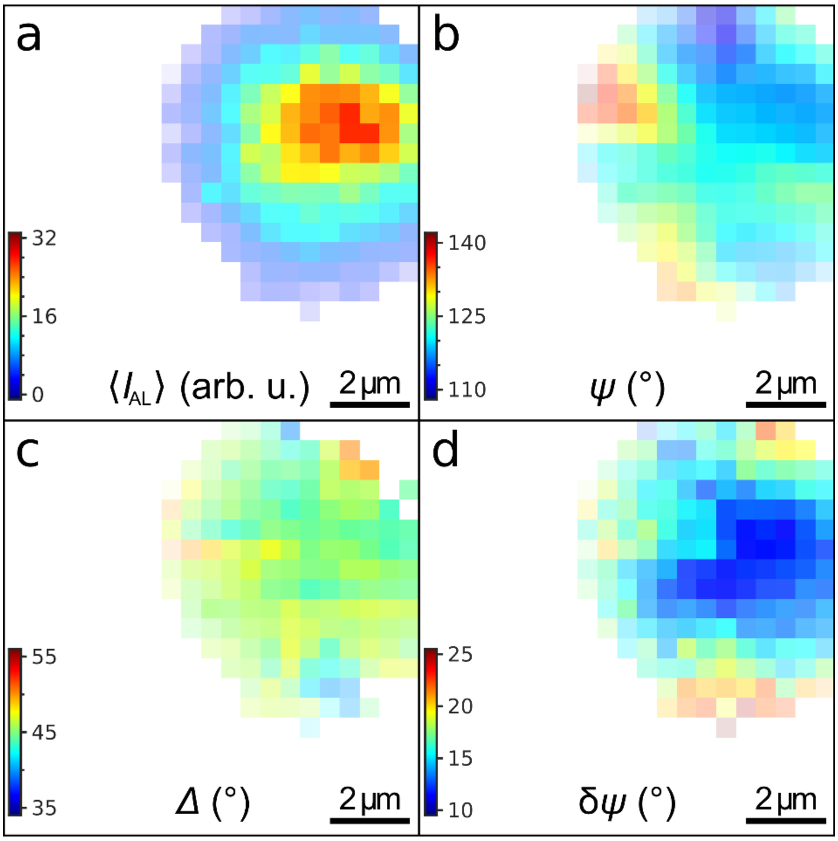

Supplementary Fig. 27. Extracted atomic lattice parameters: (a) Average intensity of the WAXS Bragg peaks $<I_{AL}>$; (b) azimuthal position ψ of the 100_{AL} crystallographic directions of the NCs; (c) the relative angle Δ between the $[010]_{AL}$ axis and the mean line **M** between the $\mathbf{a_1}$ and $\mathbf{a_2}$ basic vectors of the SL; (d) FWHM $\delta\psi$ of the angular disorder of the NCs around the mean azimuthal position ψ. The pixel size is 500 nm.

The calculated unit cell parameter of the atomic lattice from the *q*-values of the WAXS Bragg peaks is shown in Supplementary Fig. 28. It doesn't show any correlation with the spatial position within the supercrystal and remains constant at the value of a_{AL} = 0.576±0.002 nm.

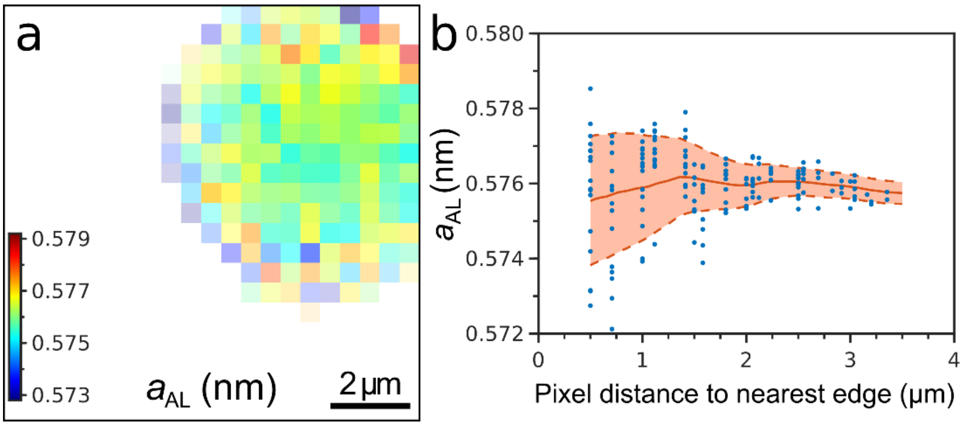

Supplementary Fig. 28. (a) Calculated unit cell parameter a_{AL} of the pseudo-cubic atomic lattice of the NCs and (b) the same value for each pixel against the distance from this pixel to the nearest edge of the supercrystal. The red line shows the mean value, the dashed lines indicate the confidence interval of $\pm\sigma$. The pixel size in a) is 500 nm.

The atomic lattice distortion was calculated from the FWHMs of the present WAXS Bragg peaks by the Williamson-Hall method (Eq. S10). The resulting values of the atomic lattice distortion are shown in Supplementary Fig. 29. The distortion gets slightly higher on the edges of the supercrystal that can be explained by the contraction of the NCs together with the superlattice. The atomic lattice distortion grows from about 0.5% in the middle of the supercrystal up to about 2.5% on the edges.

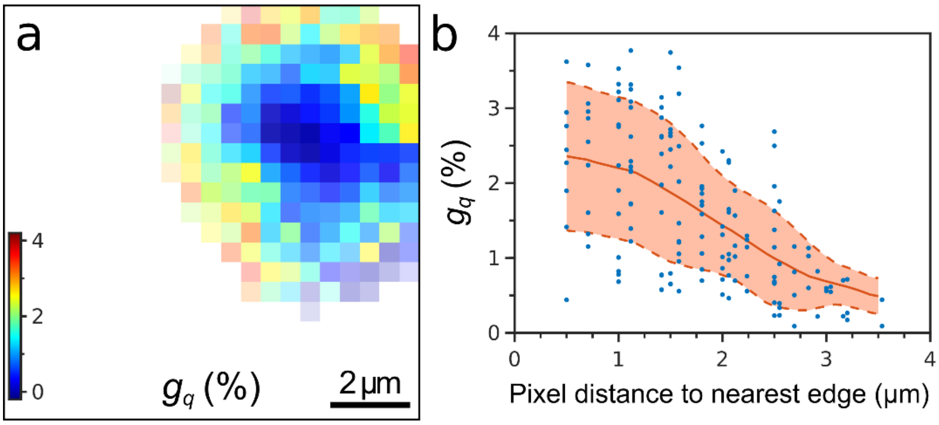

Supplementary Fig. 29. (a) Atomic lattice distortion g_q extracted from the radial FWHMs of the WAXS Bragg peaks by the Williamson-Hall method and (b) the same value for each pixel against

the distance from this pixel to the nearest edge of the supercrystal. The red line shows the mean value, the dashed lines indicate the confidence interval of $\pm\sigma$. The pixel size in (a) is 500 nm.

Supplementary Note 8. DFT-computed structures

The computation protocol used in this work is described in the main text. Cartesian coordinates of all computed structures can be accessed from Supplementary Data1.

Supplementary References

1. Baranov, D., Toso, S., Imran, M. & Manna, L. Investigation into the Photoluminescence Red Shift in Cesium Lead Bromide Nanocrystal Superlattices. *J. Phys. Chem. Lett.* **10**, 655–660 (2019).
2. Fang, Y., Wei, H., Dong, Q. & Huang, J. Quantification of re-absorption and re-emission processes to determine photon recycling efficiency in perovskite single crystals. *Nat. Commun.* **8,** 14417 (2017).
3. Zhang, M. *et al.* Growth and characterization of all-inorganic lead halide perovskite semiconductor $CsPbBr_3$ single crystals. *Cryst. Eng. Comm.* **19,** 6797–6803 (2017).
4. Williamson, G.K. & Hall, W.H. X-ray line broadening from filed aluminium and wolfram. *Acta Metall. Mater.* **1,** 22–31 (1953).
5. Langford, J. I. & Wilson, A. J. C. Scherrer after sixty years: A survey and some new results in the determination of crystallite size. *J. Appl. Cryst.* **11,** 102–113 (1978).
