# Supplementary material for: Spatially resolved fluorescence of caesium lead halide perovskite supercrystals reveals quasi-atomic behavior of nanocrystals
Source: Nat Commun. 2022 Feb 16;13:892. doi: 10.1038/s41467-022-28486-3 (PMC8850480; doi:10.1038/s41467-022-28486-3)
Supplement: Supplementary file 2 — Description of Additional Supplementary Files [file 41467_2022_28486_MOESM2_ESM.pdf]

Description of Additional Supplementary Files

File Name: Supplementary Data 1
Description: Cartesian coordinates of a CsPbBr2Cl nanocrystal computed by density functional theory.
